# Metabolic Exchange and Energetic Coupling between Nutritionally Stressed Bacterial Species: Role of Quorum-Sensing Molecules

**DOI:** 10.1128/mBio.02758-20

**Published:** 2021-01-19

**Authors:** David Ranava, Cassandra Backes, Ganesan Karthikeyan, Olivier Ouari, Audrey Soric, Marianne Guiral, María Luz Cárdenas, Marie Thérèse Giudici-Orticoni

**Affiliations:** a CNRS, Aix-Marseille University, Bioenergetic and Protein Engineering Laboratory, Mediterranean Institute of Microbiology, Marseille, France; b Aix-Marseille University, CNRS, UMR 7273, ICR, Marseille, France; c Aix-Marseille University, CNRS, Centrale Marseille, M2P2, Marseille, France; CEH–Oxford

**Keywords:** AI-2, consortium, metabolism, microbial communities, quorum sensing

## Abstract

Formation of multispecies communities allows nearly every niche on earth to be colonized, and the exchange of molecular information among neighboring bacteria in such communities is key for bacterial success. To clarify the principles controlling interspecies interactions, we previously developed a coculture model with two anaerobic bacteria, Clostridium acetobutylicum (Gram positive) and Desulfovibrio vulgaris Hildenborough (Gram negative, sulfate reducing). Under conditions of nutritional stress for *D. vulgaris*, the existence of tight cell-cell interactions between the two bacteria induced emergent properties. Here, we show that the direct exchange of carbon metabolites produced by C. acetobutylicum allows *D vulgaris* to duplicate its DNA and to be energetically viable even without its substrates. We identify the molecular basis of the physical interactions and how autoinducer-2 (AI-2) molecules control the interactions and metabolite exchanges between C. acetobutylicum and *D. vulgaris* (or Escherichia coli and *D. vulgaris*). With nutrients, *D. vulgaris* produces a small molecule that inhibits *in vitro* the AI-2 activity and could act as an antagonist *in vivo*. Sensing of AI-2 by *D. vulgaris* could induce formation of an intercellular structure that allows directly or indirectly metabolic exchange and energetic coupling between the two bacteria.

## Abstract (precis)

Bacteria have usually been studied in single culture in rich media or under specific starvation conditions. However, in nature they coexist with other microorganisms and build an advanced society.

## INTRODUCTION

Microbial communities are ubiquitous and exert a large influence in geochemical cycles and health (1–4). In natural environments, stress factors such as nutrient deficiencies and the presence of toxic compounds can induce interactions between microorganisms from the same or different species and the establishment of communities which can occupy ecological niches otherwise inaccessible to the isolated species (5, 6). Interactions between microorganisms can affect the behavior of the community either positively or negatively (7).

For studying ecological communities, it is crucial to understand how the different members communicate with each other and how this communication is regulated. Interactions may occur either by release of molecules into the environment (8) or by direct contact between the microorganisms through structures such as nanowires (9) or nanotubes (10). Dubey and coworkers were the first to demonstrate a contact-dependent exchange of cytoplasmic molecules via nanotubes in Bacillus subtilis which contributes to proper colony (11, 12). The evolution of how metabolites came to be transferred between bacteria and its functioning today were both well described (13).

The type and extent of nutritional interactions between microbes partly determine the metabolism of an entire community in a given environment (14). Very little is known about the molecular basis of interactions between species, since this is difficult to investigate, especially in nature, on account of community complexity. The use of a synthetic microbial ecosystem has considerable interest because the reduced complexity means that the investigation is more manageable, allowing not only identification of the specific community response but also description of the different events at the molecular and cellular level (15).

To further investigate interactions between bacterial species, we developed a synthetic microbial consortium constituted by two species: C. acetobutylicum (Gram positive) and D. vulgaris (Gram negative, sulfate reducing). Both organisms are involved in anaerobic digestion of organic waste matter (16, 17). Glucose, a substrate that cannot be used by *D. vulgaris* (16), is the sole carbon source in this synthetic consortium. Under this condition, the consortium produces three times more H_2_ than C. acetobutylicum alone; moreover, *D. vulgaris* is able to grow even in the absence of sulfate, its final electron acceptor for the respiration process (18). Although *D. vulgaris* can ferment lactate, a metabolite produced by C. acetobutylicum, this process is greatly inhibited by high H_2_ concentrations, preventing *D. vulgaris* from growing in the absence of methanogens (19). We observed a form of bacterial communication between adjacent cells of both types of bacteria by cell-cell interaction, under conditions of nutritional stress, with exchange in both directions of cell material, which is associated with the modification of the metabolism (18). In some cases, the interactions between C. acetobutylicum and *D. vulgaris*, resembled those described by Dubey and Ben-Yehuda (10). Moreover, these types of cell-cell interactions have also been seen in other systems, giving support to their existence and functionality (10, 13).

Nutritional stress appears crucial to induce physical contact between bacteria, since this interaction was prevented by the presence of lactate and sulfate, nutrients of *D. vulgaris.* Furthermore, Pande et al. (20) in a synthetic coculture of E. coli and Acinetobacter baylyi, after the depletion of amino acids such as histidine and tryptophan by genetic manipulation, observed nanotubular structures between the auxotrophs allowing cytoplasmic exchange. As in our case, the communication between the mutants was prevented by the presence of the nutrients. The formation of nanotubes between amino acid-starved bacteria might be a strategy to survive under amino acid-limiting conditions (13). Further evidence for the role of cell-cell connections to exchange nutrients can be found in these reviews (21–23).

Altogether, these studies suggest that for some species, cell-cell interaction (either by tight cell junctions, nanotube formation, vesicle chains, or flagella) can allow them to overcome nutrient starvation and that many materials, from small molecules to proteins or plasmids, can be passed from one cell to another. However, this requires an energetic investment to not only establish the connecting structures but also to find the suitable partners. Several questions arise. What sorts of signals are involved? What is the molecular mechanism? The fact that in several cases a nutritional stress induces interaction, but addition of nutrients prevents it, raises the question of whether there is a distress signal that is released from the starving bacteria, and another (a quenching factor) when nutrients are present? Specific signaling between cells is of great importance in the proper development of the community and in its stability in the long term (24).

Here, we partially answer these questions; in particular, we examine whether the nutritional stress, which appears to be necessary, is also sufficient. We investigated the possible role that quorum-sensing (QS) molecules could play in attaching the two bacterial cells involved in the consortia previously studied—D. vulgaris/C. acetobutylicum or D. vulgaris/E. coli—and we examined how satisfactory the energetic state of *D. vulgaris* is in the coculture when it is deprived of sulfate and why the presence of nutrients prevents interaction between these bacteria.

## RESULTS

### Tight bacterial interaction in the coculture allows *D. vulgaris* to be metabolically active and to grow by using carbon metabolites produced by *C. acetobutylicum*.

**FIG 1:**
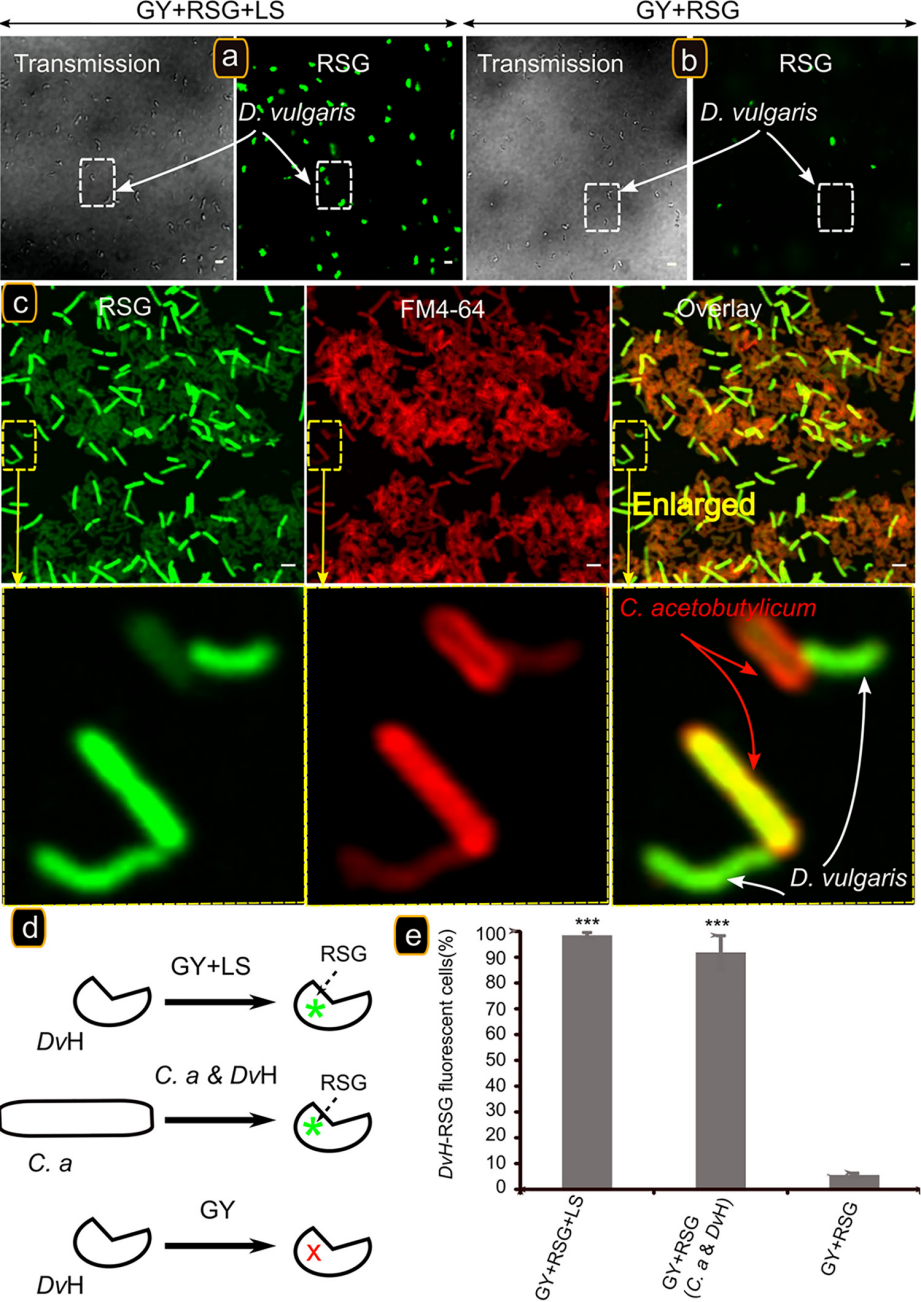
The presence of C. acetobutylicum is required for energetic activation of *D. vulgaris*. *D. vulgaris* growing exponentially in Starkey medium was washed twice and starved by incubation in GY medium for 20 h at 37°C. A starved culture was divided into three subcultures and supplemented with 1 μM RSG (final concentration). The first was activated with 10 mM lactate/sulfate (LS) (a), and the second remained starved (b). The two subcultures were sampled and visualized by fluorescence confocal microscopy after 20 h of incubation at 37°C. The third subculture was mixed with C. acetobutylicum cells. (c) After 20 h of incubation at 37°C, the culture was sampled, left for 5 min in contact with FM4-64 (in order to visualize the two strains), and visualized by fluorescence confocal microscopy. Scale bar, 2 μm (all panels). (d) Schematic representation of RSG activation or not in the cell under different conditions. The percentages of *D. vulgaris* RSG-fluorescent cells in GY medium supplemented with lactate and sulfate, in GY medium with C. acetobutylicum, and in GY medium in pure culture (E) are shown. The data represent means ± the standard deviations (SD; *n* = 3) compared to *D. vulgaris* in a pure culture in GY medium. *P* values were calculated using Tukey HSD tests (* *P* < 0.05; ** *P* < 0.01; *** *P* < 0.001). Abbreviations: *C.a*, Clostridium acetobutylicum; *Dv*H, Desulfovibrio vulgaris Hildenborough. The microscopy fields analyzed were obtained from 3 biological replicates obtained independently containing between 100 and 150 cells.

Based on metabolic and microscopic experiments, our previous results demonstrated that conditions of nutritional stress of D. vulgaris induce a tight interaction between D. vulgaris and C. acetobutylicum, in coculture, which allows the exchange of cytoplasmic molecules and the growth of D. vulgaris (see Fig. 1 in reference 18). If the tight interaction is prevented when either D. vulgaris or C. acetobutylicum are confined in a dialysis tube, *D. vulgaris* cannot grow (18). A similar phenomenon was observed between D. vulgaris and E. coli DH10B (18) (see Fig. S1 in the supplemental material). This growth suggests an adequate energetic state of *D. vulgaris* in coculture, despite of the lack of sulfate, its final electron acceptor, and shows that it can use metabolites from C. acetobutylicum, since *D. vulgaris* cannot use glucose (16).

10.1128/mBio.02758-20.1 FIG S1 Selected images showing structures connecting the two bacteria. *D. vulgaris* growing exponentially in Starkey medium was labeled with calcein, washed with GY medium, and mixed with unlabeled E. coli strains DH10B. After 20 h of incubation at 37°C, the culture was sampled, left for 5 min in contact with FM4-64 (in order to visualize the two strains), and visualized by fluorescence confocal microscopy. Red arrows indicate apparent connections between bacterial cells. Select images showing the formation of possible tubular protrusions bridging neighboring cells are visible on the surface *D. vulgari*s (right panel). Scale bar, 2 μm (all panels). Download FIG S1, TIF file, 2.5 MB. Copyright © 2021 Ranava et al. 2021 Ranava et al. This content is distributed under the terms of the Creative Commons Attribution 4.0 International license.

To evaluate the physiological impact that C. acetobutylicum has on *D. vulgaris* in coculture, we labeled *D. vulgaris* cells with RedoxSensor Green (RSG), a small molecule that can easily pass through the membranes of Gram-negative and Gram-positive bacteria, used as a respiration sensor to identify metabolically active cells. It has been tested on numerous bacteria, as an indicator of active respiration in pure or cocultures (9, 25). If *D. vulgaris* became metabolically active due to its physical interaction with C. acetobutylicum, then an RSG fluorescence should be detected as for *D. vulgaris* cultivated in glucose-yeast extract (GY) medium supplemented by lactate and sulfate (respiration) (Fig. 1a). As expected, under respiration conditions, i.e., in the presence of lactate and sulfate, substrates of *D. vulgaris*, *D. vulgaris* in pure culture grows well, and all the cells show intense RSG fluorescence, in contrast to culture in GY medium, where there is no growth and very few cells fluoresce (Fig. 1a and b). As a control, we added CCCP (carbonyl cyanide *m*-chlorophenylhydrazone), which by dissipating the proton gradient and abolishing the ATP synthesis, tightly impacts RSG fluorescence (see Fig. S2). When *D. vulgaris* is labeled with RSG, as described above, and then cultivated for 20 h with C. acetobutylicum in GY medium, despite the lack of sulfate it shows a significant RSG fluorescence indicating that the cells have enough reducing power to reduce redox green and are metabolically active (Fig. 1c to e). Furthermore, C. acetobutylicum, which was not labeled by RSG at the beginning, also fluoresces intensely, indicating that RSG has been transferred from *D. vulgaris*.

10.1128/mBio.02758-20.2 FIG S2 Effect of electron transport chain uncoupler on RedoxSensor Green fluorescence. *D. vulgaris* growing exponentially in Starkey medium was washed twice and starved by incubation in GY medium for 20 h at 37°C. A starved culture was diluted in GY medium (5 ml) containing 10 mM lactate and sulfate and supplemented with 1 μM RSG without or with 10 μM uncoupler CCCP (final concentrations). The culture was sampled and visualized by fluorescence confocal microscopy after 20 h of incubation at 37°C. Scale bar, 2 μm (all panels; *n* = 3). Download FIG S2, TIF file, 1.0 MB. Copyright © 2021 Ranava et al. 2021 Ranava et al. This content is distributed under the terms of the Creative Commons Attribution 4.0 International license.

**FIG 2:**
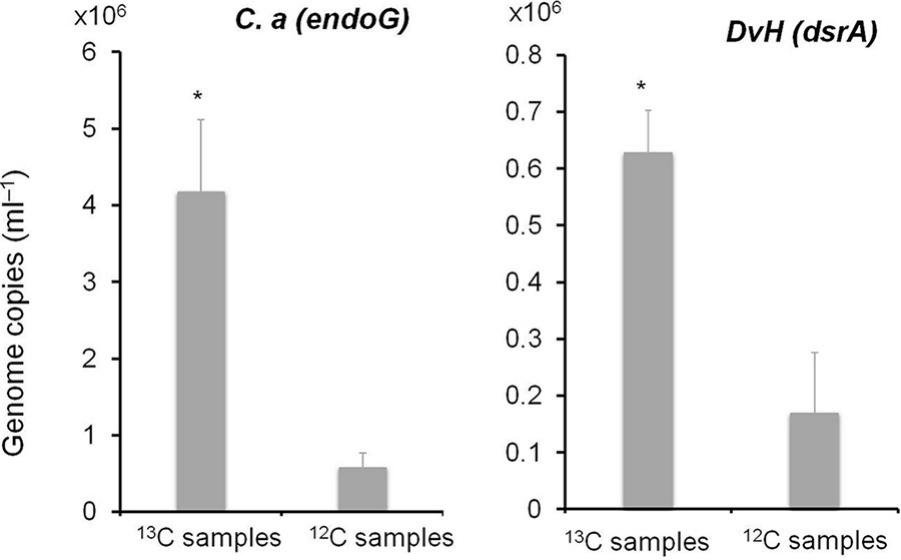
Quantification of *D*. *vulgaris* (*dsrA*) and C. acetobutylicum (*endoG*) on fractions (corresponding to fraction 28 in Fig. S3 in the supplemental material) containing ^13^C from C. acetobutylicum ^13^C plus *Dv*H ^12^C in [^13^C]glucose medium DNA and on corresponding fractions containing ^12^C from C. acetobutylicum ^12^C plus *Dv*H ^12^C in [^12^C]glucose medium DNA. The data represent means ± the SD (*n* = 3) compared to ^12^C samples. *P* values were calculated using Tukey HSD tests (*, *P* < 0.05; **, *P* < 0.01; ***, *P* < 0.001). Abbreviations: *C.a*, Clostridium acetobutylicum; *Dv*H, Desulfovibrio vulgaris Hildenborough.

To investigate carbon exchange between the two bacteria, we used Stable Isotope Probing, growing C. acetobutylicum, either alone or in coculture with unlabeled *D. vulgaris*, on [^13^C]glucose medium. Total DNA was extracted from cells collected at the end of the exponential phase, and [^13^C]DNA (heavier) and [^12^C]DNA (lighter) were separated by density gradient centrifugation and examined on an agarose gel (see Fig. S3 in the supplemental material). Analysis of the different fractions using specific gene markers for the two bacteria shows that in the coculture DNA from *D. vulgaris* is “heavy” (^13^C labeled), indicating that metabolites derived from [^13^C]glucose were transferred between the two bacteria and used by *D. vulgaris*, despite the absence of sulfate. Quantitative PCR emphasized the presence of *D. vulgaris* ^13^C-labeled DNA with C. acetobutylicum ^13^C-labeled DNA in the same fraction (Fig. 2). A small amount of ^12^C-unlabeled DNA (from the two bacteria) can be detected in this high-density fraction when an unlabeled coculture is used, but it was not significant in relation to the total DNA and probably in line with the initial *D. vulgaris* inoculum. Since *D. vulgaris* cannot grow on glucose or other hexoses, the ^13^C-labeled DNA from *D. vulgaris* must have been formed using metabolites produced by C. acetobutylicum.

10.1128/mBio.02758-20.3 FIG S3 Analysis of fractions from density gradient on agarose gel. Fractions of C. acetobutylicum ^12^C plus *D. vulgaris* ^12^C in [^12^C]glucose medium DNA as a negative control (a) and C. acetobutylicum ^13^C plus *D. vulgaris* ^12^C in [^13^C]glucose medium DNA (b). Fractions A and B were centrifuged together. Fractions 27 to 30 contained [^13^C]DNA (heavier) and fractions 33 to 35 contained [^12^C]DNA (lighter). Download FIG S3, TIF file, 1.3 MB. Copyright © 2021 Ranava et al. 2021 Ranava et al. This content is distributed under the terms of the Creative Commons Attribution 4.0 International license.

### *C. acetobutylicum* produces AI-2.

**FIG 3:**
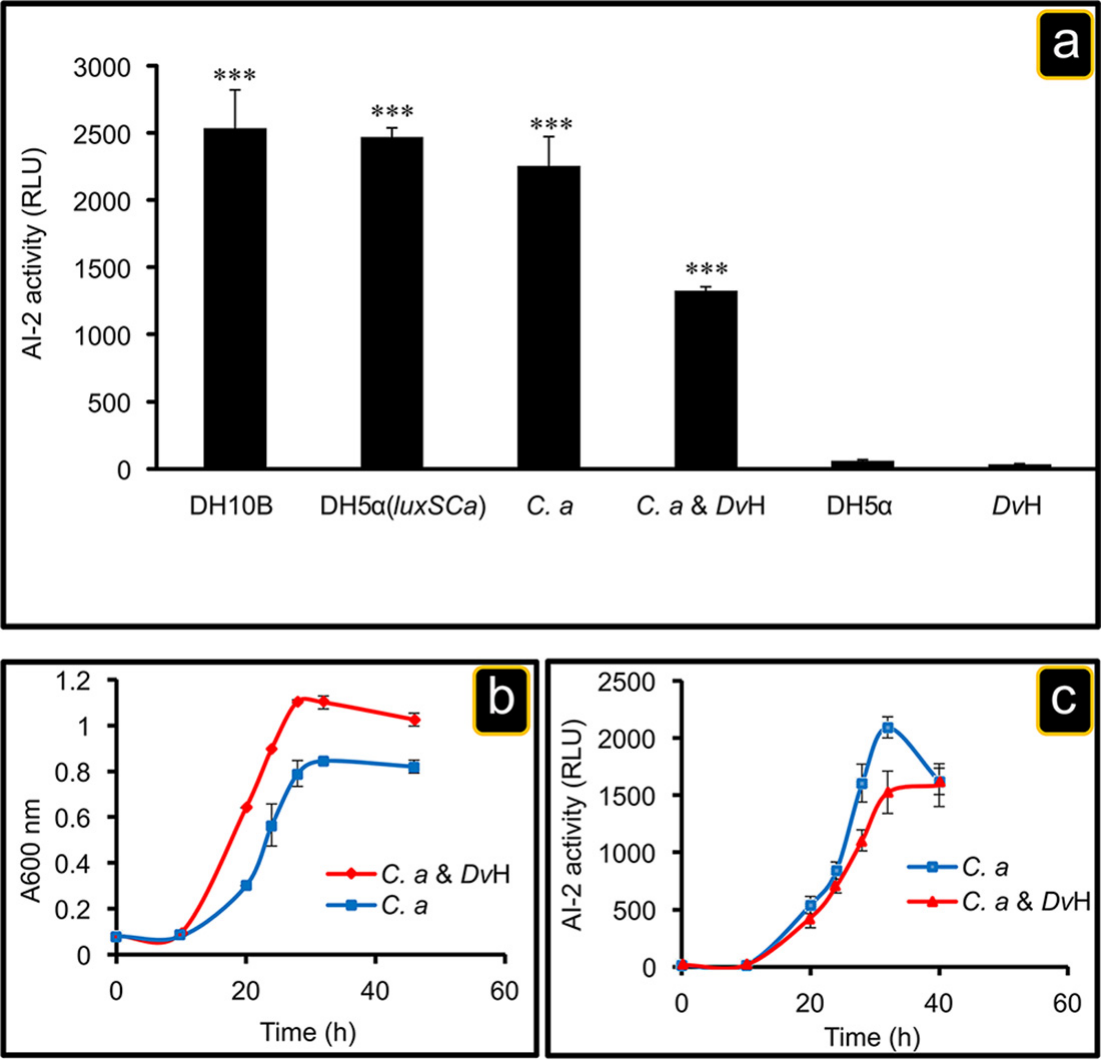
Extracellular AI-2 activity. (a) From left to right, a pure culture of E. coli DH10B (AI-2 producer, positive control), a pure culture of *E. coli* DH5α expressing the *luxS_Ca_* gene of C. acetobutylicum, a pure culture of C. acetobutylicum, a coculture of C. acetobutylicum and *D. vulgaris*, and a pure culture of E. coli DH5α (AI-2 nonproducer, negative control), all grown in GY medium to exponential phase (i.e., OD_600_ ≈ 0.8). For the right extreme, a *D. vulgaris* wild-type strain was grown in Starkey medium. Aliquots of different cultures were obtained at exponential phase and filtered to remove cells. The AI-2 activity in the cell culture supernatant was measured using a V. harveyi BB170 bioassay as described in Materials and Methods. The data represent means ± the SD (*n* = 3) compared to the extracellular AI-2 activity from the *D. vulgaris* wild type. *P* values were calculated using Tukey HSD tests (*, *P* < 0.05; **, *P* < 0.01; ***, *P* < 0.001). (b and c) Time courses of extracellular AI-2 accumulation in a pure culture of C. acetobutylicum or in a coculture of C. acetobutylicum and *D. vulgaris*. Exponentially growing C. acetobutylicum in 2YTG medium and *D. vulgaris* in Starkey medium under anaerobic conditions were washed twice with fresh GY medium. Next, C. acetobutylicum was inoculated alone or mixed with *D. vulgaris* into GY medium at time zero, and aliquots were obtained at the indicated times. Cell growth was monitored by measuring the absorbance at 600 nm (b), and the AI-2 activity in cell-free culture fluids was measured in a pure culture of C. acetobutylicum or in coculture using a V. harveyi bioluminescence assay (c). The AI-2 activity is reported as relative light units (RLU) of BB170 bioluminescence. Abbreviations: *C.a*, Clostridium acetobutylicum; D*v*H, Desulfovibrio vulgaris Hildenborough; DH5α, Escherichia coli DH5α; DH5α(*luxS_Ca_*), Escherichia coli DH5α expressing the *luxS_Ca_* gene; DH10B, Escherichia coli DH10B.

As nutritional restrictions of *D. vulgaris* appear indispensable for inducing physical interactions between C. acetobutylicum and *D. vulgaris*, we investigated whether in addition to being necessary they were also sufficient, or if another element, such as QS molecules, which are often associated with bacterial communication (26, 27), was required under our conditions. Since the coculture is composed of an association of Gram-positive and Gram-negative bacteria, autoinducer-2 (AI-2), known to be involved in interspecies communication, appears as a good candidate to be tested (28). AI-2 is widely accepted as the universal cell-to-cell signal in prokaryotic microorganisms (29, 30). *Clostridium* species are known to develop QS systems based on peptides, but QS remains relatively unknown in sulfate-reducing bacteria (SRB), although inferences on the presence of putative QS systems in them can be made (31) and, more recently, a role for QS (AHL molecules) in *D. vulgaris* biofilm formation, electron transfer, and metabolism was proposed (32). However, no AI-2 signaling/sensing had been described in C. acetobutylicum or *D. vulgaris*. To determine whether C. acetobutylicum could generate AI-2-like activity, a cell-free supernatant of exponential-phase C. acetobutylicum culture was tested for its ability to induce luminescence in the Vibrio harveyi BB170 AI-2 reporter strain. This cell-free supernatant stimulated luminescence in a similar manner to cell-free supernatant of exponential-phase E. coli DH10B (AI-2 producer) culture (Fig. 3a).

Furthermore, AI-2 activity was also detected in the sterile-filtered culture supernatant of an exponential-phase C. acetobutylicum and D. vulgaris coculture. The last step of the AI-2 biosynthetic pathway is catalyzed by the *luxS* gene product (33), which is present in the C. acetobutylicum genome (CA_C2942) annotated as *S*-ribosylhomocysteinase and could encode the LuxS protein. Genetic engineering on genus *Clostridium* remains difficult and requires the utilization of specific genetic tools. To avoid this and to test whether this gene is involved in AI-2 production, the putative *luxS* gene from C. acetobutylicum (*luxS_Ca_*) was introduced into E. coli DH5α, which does not produce AI-2 due to a 60-amino-acid deletion stemming from a 1-bp deletion resulting in early truncation of *luxS* (formerly *ygaG*.) (34). E. coli DH5α is commonly used as negative control for AI-2 production in different bacterial strains (34–36). As hypothesized, the cell-free supernatant of exponential-phase E. coli DH5α (*luxS_Ca_*) culture, expressing the *luxS_Ca_* gene of C. acetobutylicum, has AI-2 activity (Fig. 3a). In contrast, *D. vulgaris* does not have a homolog of the *luxS* gene, and the cell-free culture supernatant collected from an exponential-phase *D. vulgaris* culture, grown in Starkey medium, does not have AI-2 activity. This finding agrees with what is observed with the cell-free culture supernatant of E. coli DH5α (Fig. 3a). Since *D. vulgaris* does not produce AI-2, the AI-2 molecules present in the coculture are likely to be produced by C. acetobutylicum. To verify whether C. acetobutylicum AI-2 production follows the growth, cell-free culture supernatants from C. acetobutylicum or from a C. acetobutylicum/*D. vulgaris* coculture taken at different times were used in a V. harveyi bioluminescence assay, as described in Materials and Methods. As shown in Fig. 3b and c, C. acetobutylicum can synthesize functional AI-2 molecules, the synthesis following the growth in single culture, as well as in coculture, indicating that its production is independent of the presence of *D. vulgaris*.

### Cytoplasmic exchanges of molecules between bacteria in the coculture, as well as the metabolic activity of *D. vulgaris*, depend on the presence of AI-2.

**FIG 4:**
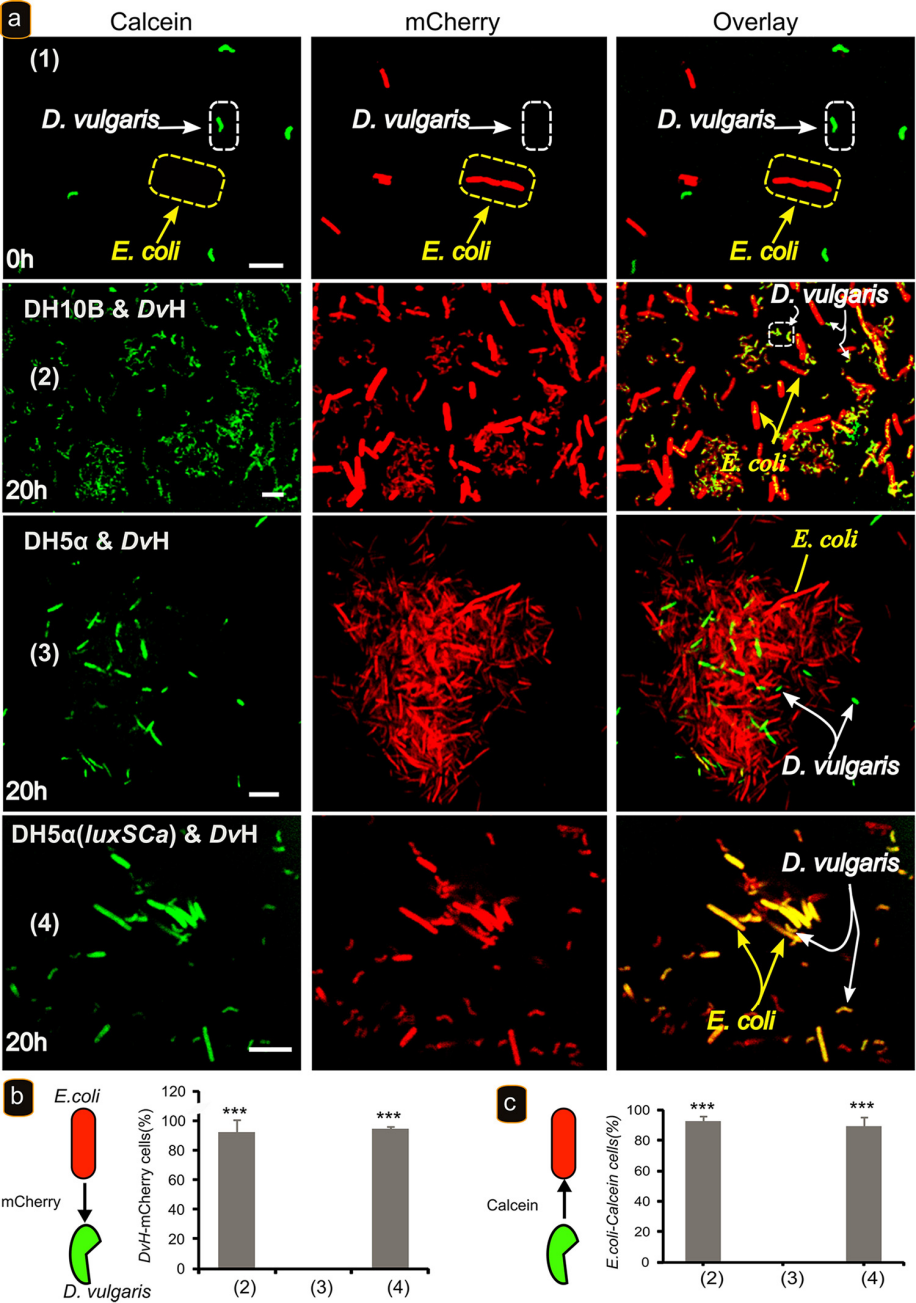
AI-2 is required for cytoplasmic molecule exchange between *D. vulgaris* and E. coli. (a) *D. vulgaris* growing exponentially (OD_600_ of 0.6) in Starkey medium was labeled with calcein, washed with GY medium, and mixed with E. coli strains DH10B (panel 1) at zero time, DH10B (panel 2), DH5α (panel 3), and DH5α(*luxS_Ca_*) (panel 4) labeled with mCherry and visualized by fluorescence confocal microscopy at time zero (panel 1) or after 20 h of incubation at 37°C (panels 2 to 4) in GY medium. Scale bar, 2 μm (all panels). (b) Percentage of *D. vulgaris* cells that acquired mCherry when cocultured with different strains of E. coli. (c) Percentage of E. coli cells that acquired calcein when cocultured with *D. vulgaris*. The data represent means ± the SD (*n* = 3) compared to E. coli DHα (columns 3). *P* values were calculated using Tukey HSD tests (*, *P* < 0.05; **, *P* < 0.01; ***, *P* < 0.001). Abbreviations: *Dv*H, D. vulgaris Hildenborough; DH5α, E. coli DH5α; DH5α(*luxS_Ca_*), E. coli DH5α expressing the *luxS_Ca_* gene; DH10B, E. coli DH10B. The microscopy fields analyzed were obtained from three biological replicates obtained independently and containing between 100 and 150 cells.

That C. acetobutylicum and E. coli DH10B, used in previous studies (18), both produce AI-2 raised a question regarding a situation where AI-2 was not present, that is, if E. coli DH5α, were used. So, E. coli DH5α or E. coli DH10B, both harboring the pRSET-B mCherry plasmid containing the gene *mCherry*, was mixed with *D. vulgaris* cells lacking the *mCherry* gene but labeled with calcein or not, and the coculture was analyzed by microscopy. Calcein-acetoxymethyl (AM) ester is a small nonfluorescent derivative of calcein that is sufficiently hydrophobic to pass readily through cell membranes. Once inside, the AM group is cleaved by esterases, yielding the more hydrophilic calcein (623 Da), which is unable to cross membranes and is sequestered in the cytoplasm. The loss of the AM group also enables calcein to readily bind intracellular calcium, resulting in a strong yellowish-green fluorescence. When *D. vulgaris* cells were cultivated on GY medium with E. coli DH10B, more than 90% of *D. vulgaris* cells acquired a mCherry fluorescence signal after 20 h of culture (Fig. 4a, panel 2; Fig. 4b, column 2; and Fig. S4, panel 1).

10.1128/mBio.02758-20.4 FIG S4 AI-2 is required for mCherry transfer from E. coli to *D. vulgaris*. *D. vulgaris* Hildenborough (*Dv*H) growing exponentially in Starkey medium was washed in GY medium, and mixed E. coli strains DH10B, DH5α, and DH5α(*luxS_Ca_*) were labeled with mCherry and visualized by fluorescence confocal microscopy after 20 h incubation at 37°C in GY medium. Scale bar, 2 μm (all panels). The data represent means ± the SD (*n* = 3) compared to *D. vulgaris* cocultured with E. coli DH5α in GY medium (non-AI-2 producer, column 3). *P* values werecalculated using Tukey HSD tests (*, *P* < 0.05; **, *P* < 0.01; ***, *P* < 0.001). Abbreviations: *Dv*H, D. vulgaris Hildenborough; DH5α, E. coli DH5α; DH5α(*luxS_Ca_*), E. coli DH5α expressing the *luxS_Ca_* gene; DH10B, E. coli DH10B. Download FIG S4, TIF file, 2.9 MB. Copyright © 2021 Ranava et al. 2021 Ranava et al. This content is distributed under the terms of the Creative Commons Attribution 4.0 International license.

In contrast, no mCherry fluorescence was observed in *D. vulgaris* cells when they were cultivated with E. coli DH5α (Fig. 4a, panel 3; Fig. 4c, column 3; and Fig. S4, panel 3). However, *D. vulgaris* cells (around 90%) became mCherry-fluorescent when they are cocultured with E. coli DH5α expressing *luxS_Ca_* gene (Fig. 4a, panel 4; Fig. 4b, column 3; and Fig. S3, panel 2).

Taken together, these results suggest that AI-2 is essential for cell-to-cell communication and, in consequence, the exchange of cytoplasmic molecules in coculture, but it is not sufficient, since nutritional stress is also required. Moreover, these results confirm that *D. vulgaris* is able to survive even when the interactions were inhibited as proposed earlier (18). These results may explain why Pande et al. (20) reported that nanotubes, used to transfer amino acids, were observed between E. coli auxotroph mutants and between E. coli and Acinetobacter baylyi mutants, since in both cases there is the possibility of AI-2 produced by E. coli. In contrast, no nanotubes were observed between mutants of A. baylyi in which *luxS* is absent, in agreement with our genome bioinformatic analysis. Thus, when AI-2 is not produced, there may be no physical interaction even if there is a nutritional stress.

**FIG 5:**
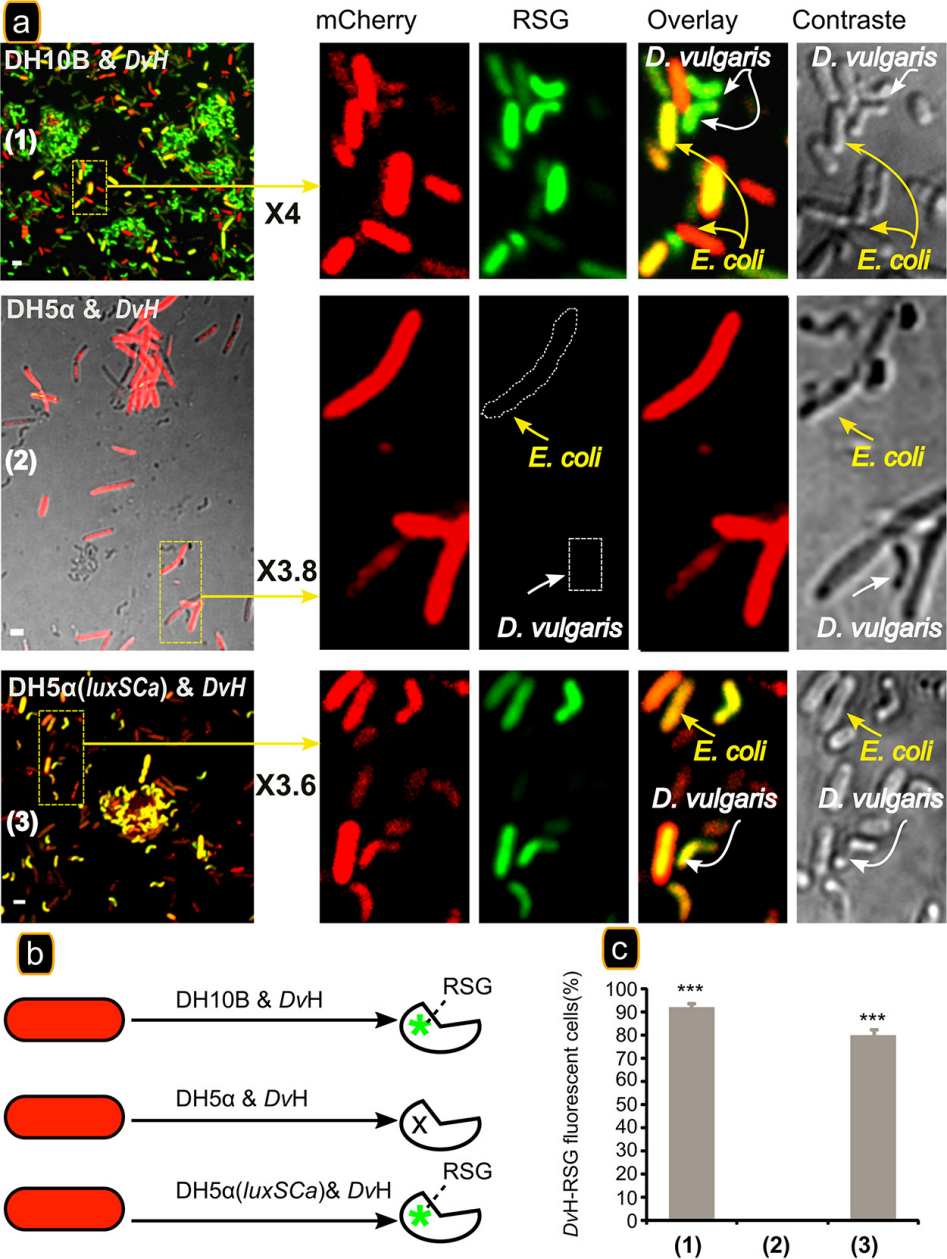
AI-2 is required for energetic activation of *D. vulgaris*. (a) *D. vulgaris* was grown exponentially in Starkey medium and was then washed twice and starved by incubation in GY medium for 20 h at 37°C. A starved culture was divided into three subcultures and supplemented with 1 μM RSG (final concentration). The subcultures were incubated at 37°C for 1 h, and then E. coli strains DH10B (panel 1), DH5α (panel 2), and DH5α(*luxS_Ca_*) (panel 3) labeled with mCherry were added. The three subcultures were sampled and visualized by fluorescence confocal microscopy after 20 h of incubation at 37°C. Scale bar, 2 μm (all panels). (b) Schematic representation of RSG activation or not in cells under the different conditions indicated. (c) Percentages of *D. vulgaris* RSG-fluorescent cells cocultured in GY medium with E. coli strains. The data represent means ± the SD (*n* = 3) compared to *D. vulgaris* cocultured with E. coli DH5α in GY medium (non-AI-2 producer). *P* values were calculated using Tukey HSD tests (*, *P* < 0.05; **, *P* < 0.01; ***, *P* < 0.001). Abbreviations: *Dv*H, D. vulgaris Hildenborough; DH5α, E. coli DH5α; DH5α(*luxS*), E. coli DH5α expressing the *luxS* gene; DH10B, E. coli DH10B. The microscopy fields analyzed were obtained from three biological replicates obtained independently containing between 100 and 150 cells.

In view of the necessity of AI-2 to allow growth of *D. vulgaris* in the coculture, we tested its effect on the energetic state of the cells with RSG, as in Fig. 1. The lack of AI-2 should prevent RSG fluorescence in *D. vulgaris* by preventing physical interaction between E. coli and *D. vulgaris* in GY medium. *D. vulgaris* cells were incubated with RSG as described above and mixed with E. coli DH5α or E. coli DH10B harboring the gene *mCherry*, and the coculture was analyzed by microscopy after 20 h of incubation at 37°C. *D. vulgaris* cells displayed a significant RSG fluorescence (90% of the cells) when they are mixed with E. coli DH10B (Fig. 5a, panel 1, and Fig. 5c, column 1). In contrast, no RSG fluorescence was observed in *D. vulgaris* cells when cocultured with E. coli DH5α (Fig. 5a, panel 2, and Fig. 5c, column 2). Moreover, E. coli DH5α does not show RSG fluorescence as E. coli DH10B and C. acetobutylicum (Fig. 1), which supports the absence of cytoplasmic exchange. In contrast, *D. vulgaris* cells cocultivated with E. coli DH5α complemented with the *luxS_Ca_* gene displayed RSG fluorescence (about 80% of the cells) similar to that observed with E. coli DH10B (Fig. 5a, panel 3, and Fig. 5c, column 3). These results strongly support that the AI-2 molecule is important for physical interaction between *D. vulgaris* and E. coli and thus in metabolic activation of *D. vulgaris*. All of these results suggest that *D. vulgaris* can detect AI-2.

### *D. vulgaris* produces an antagonist of AI-2 in the presence of sulfate and under respiratory conditions.

The effect of AI-2 in the coculture suggests that *D. vulgaris* can detect it. However, lactate and sulfate in the coculture medium allow the growth of the two bacteria but prevent physical contact between the two bacteria and the transfer of cytoplasmic molecules (18) despite the fact that C. acetobutylicum and E. coli can produce AI-2. This suggests a regulatory mechanism linked to the presence of lactate and sulfate in the culture medium and/or to the sulfate respiration metabolism of *D. vulgaris*. At least two hypotheses may explain this. (i) In the presence of lactate and sulfate, C. acetobutylicum does not produce AI-2. (ii) D. vulgaris in the presence of sulfate produces one or several compound that interfere with AI-2 activity or its production.

**FIG 6:**
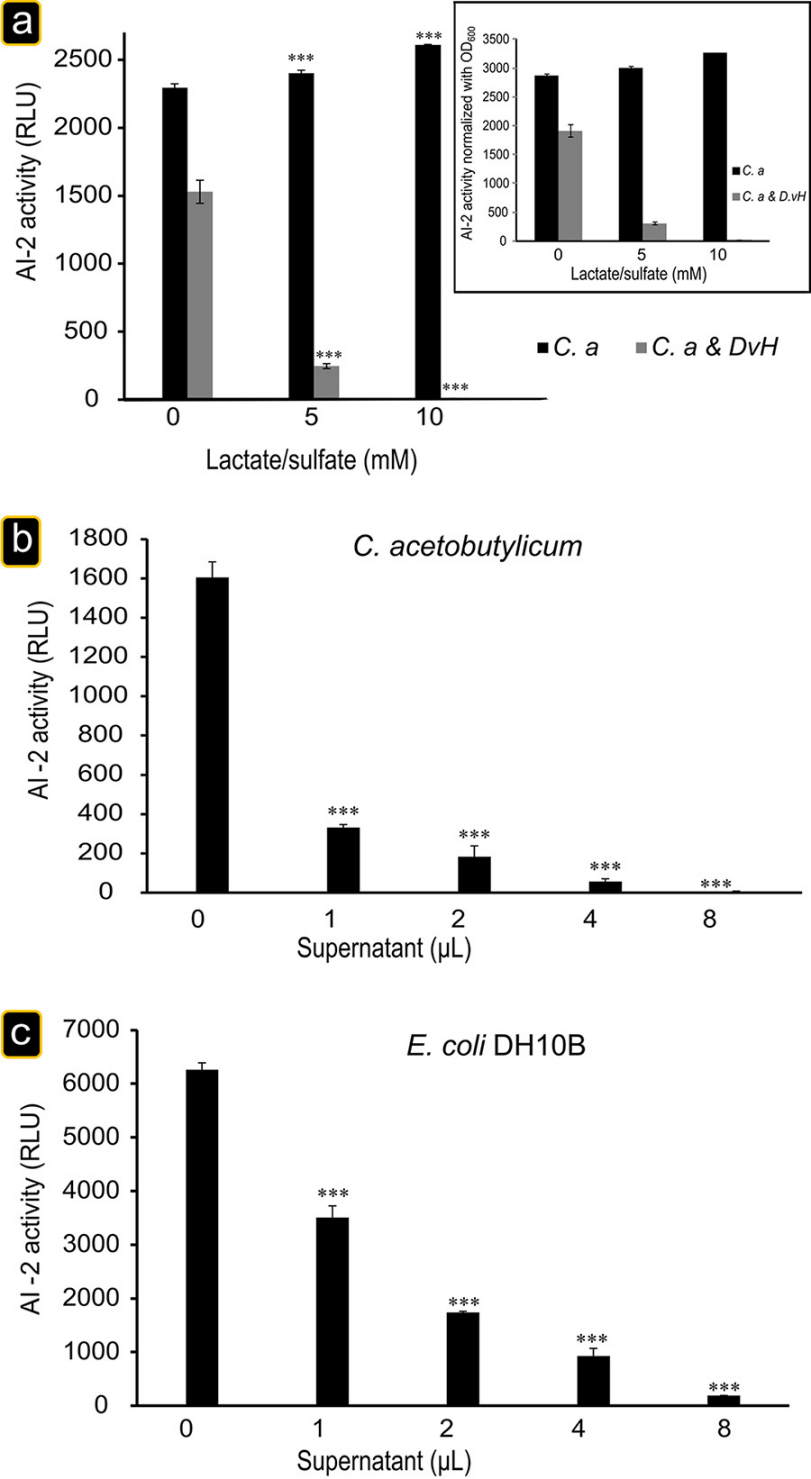
Inhibition of AI-2 activity. (a) The addition of lactate and sulfate to the coculture impaired AI-2 activity. Exponentially growing C. acetobutylicum in 2YTG medium or *D. vulgaris* in Starkey medium under anaerobic conditions was washed two times with fresh GY medium. Next, C. acetobutylicum was inoculated alone (black) or mixed with *D. vulgaris* (gray) into GY medium supplemented with increased concentrations of lactate and sulfate (5 and 10 mM). After 30 h of incubation at 37°C, the AI-2 activity was analyzed using the V. harveyi reporter strain BB170. The inset presents normalized AI-2 activity to the number of cells in the culture. *D. vulgaris* supernatant inhibits AI-2 activity. C. acetobutylicum (*C.a*) strain was grown in GY medium for 30 h at 37°C. (b) The activity of AI-2 in the filtered samples was then analyzed using V. harveyi reporter strain BB170 in the presence of various quantities (1, 2, 4, and 8 μl) of *D. vulgaris* filtered (0.2 μm) supernatant grown on Starkey medium for 30 h. AI-2 activity is reported as the RLU of BB170 bioluminescence. E. coli DH10B strain was grown in GY medium for 30 h at 37°C. (c) The activity of AI-2 in the filtered samples was then analyzed using V. harveyi reporter strain BB170 in the presence of various quantities (1, 2, 4, and 8 μl) of *D. vulgaris* filtered (0.2 μm) supernatant grown on Starkey medium for 30 h. The data represent the mean ± the SD (*n* = 3) AI-2 activity measured for C. acetobutylicum in GY medium with 5 and 10 mM lactate and sulfate compared to C. acetobutylicum in GY medium (black columns in panel a) and C. acetobutylicum plus *D. vulgaris* in GY medium with 5 and 10 mM lactate and sulfate compared to C. acetobutylicum plus *D. vulgaris* in GY medium (gray columns in panel a) compared to AI-2 activity measured for AI-2 produced by C. acetobutylicum and E. coli without *D. vulgaris* supernatant (panels b and c, respectively). *P* values were calculated using Tukey HSD tests (*, *P* < 0.05; **, *P* < 0.01; ***, *P* < 0.001). Abbreviations: *C.a*, C. acetobutylicum; *Dv*H, D. vulgaris Hildenborough.

The addition of lactate and sulfate to pure culture of C. acetobutylicum does not impair the production of AI-2 (Fig. 6a), nor C. acetobutylicum metabolism with 5 to 6 mM of butyrate produced. In contrast, in coculture with *D. vulgaris*, the AI-2 activity detected in the sterile-filtered culture supernatant (without any bacterial cells) in exponential phase greatly decreased even in the presence of low quantity of lactate and sulfate (5 mM) and was not detected by growing the coculture in the presence of 10 mM (Fig. 6a), suggesting that under sulfate respiratory conditions *D. vulgaris* could produce one or more metabolites that inhibit the activity of AI-2. To exclude the possibility that the supernatant taken from the culture grown in the presence of lactate and sulfate could inhibit the growth of the reporter strain, the growth of V. harveyi was measured. As shown in the Fig. S5 in the supplemental material, the addition of different supernatants does not affect the growth the V. harveyi.

10.1128/mBio.02758-20.5 FIG S5 The addition of cell free supernatants does not affect the growth of V. harveyi reporter strain BB170. AN overnight culture of V. harveyi (grown for 16 h in AB medium) was diluted 1/5,000 in fresh AB medium. The diluted cells (90 μl) were added to 96-well plates (Corning) containing 10 μl of filtered supernatants of a mixed culture of *D. vulgaris* and C. acetobutylicum or a pure culture of C. acetobutylicum grown in SY medium supplemented with 0, 5, and 10 mM lactate and sulfate. The microtiter plate was incubated at 30°C with shaking at 160 rpm, and the growth was measured after 5 h using a Tecan GENioS plate reader. Download FIG S5, TIF file, 0.2 MB. Copyright © 2021 Ranava et al. 2021 Ranava et al. This content is distributed under the terms of the Creative Commons Attribution 4.0 International license.

An important point is that, under these conditions, butyrate was produced (5 mM), indicating that C. acetobutylicum is metabolically active and that the lack of AI-2 is not due to a metabolic inactivity of C. acetobutylicum.

To test the presence of molecules that could interfere with AI-2 activity in sulfate respiratory conditions, we monitored the AI-2 activity present in the exponential-phase supernatant cultures of E. coli or C. acetobutylicum in the presence of increasing amounts of a *D. vulgaris* supernatant culture grown in Starkey medium for 30 h. A sterile-filtered culture supernatant of *D. vulgaris* inhibits the AI-2 activity of C. acetobutylicum (Fig. 6b) and E. coli DH10B (Fig. 6c) cell-free supernatant in a dose-dependent manner, indicating that *D. vulgaris*, in the presence of lactate and sulfate and independently of the presence of the other bacteria, released an AI-2-inhibiting compound (or a mixture of such compounds) into the culture medium. Moreover, this result confirms that the absence of AI-2 activity is not associated with the downregulation of AI-2 production.

**FIG 7:**
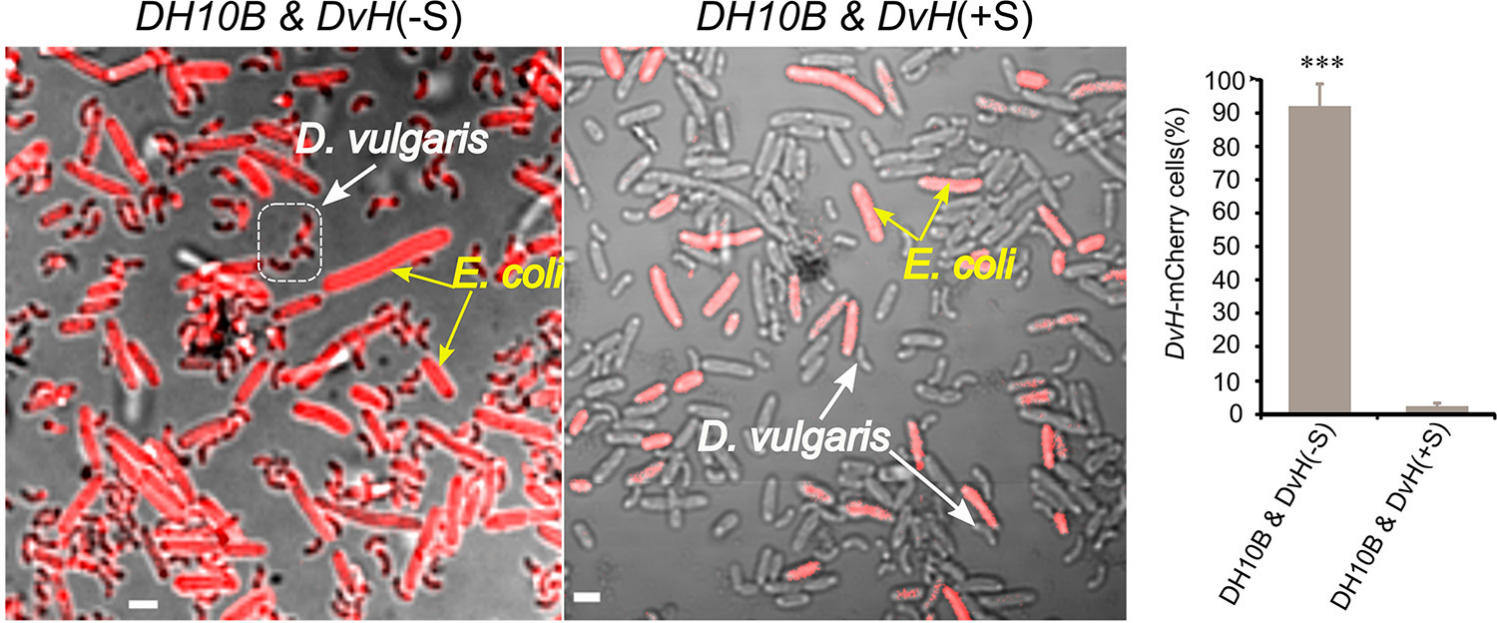
Impact of supernatant of *D. vulgaris* growing in Starkey on cytoplasmic molecule exchange. *D. vulgaris* growing exponentially in Starkey medium was washed with GY medium and mixed with E. coli DH10B (AI-2 producer) labeled with mCherry and grown in 5 ml of GY medium supplemented (+S) or not (–S) with 100 μl of *D. vulgaris* filtered (0.2 μm) supernatant grown on Starkey medium. The culture was visualized by fluorescence confocal microscopy after 20 h of incubation at 37°C. Scale bar, 2 μm (all panels). The data represent means ± the SD (*n* = 3) compared to the percentage of mCherry *D. vulgaris* fluorescent cells when cocultured with E. coli DH10B in GY medium supplemented with *D. vulgaris* Starkey supernatant. *P* values were calculated using Tukey HSD tests (*, *P* < 0.05; **, *P* < 0.01; ***, *P* < 0.001). Abbreviations: *C.a*, C. acetobutylicum; *Dv*H, D. vulgaris Hildenborough; DH10B, E. coli DH10B.

We monitored the production kinetics of the AI-2 inhibiting compound by *D. vulgaris* by taking samples of the sterile-filtered supernatant of a *D. vulgaris* culture in Starkey medium at different times. An AI-2 inhibitor is detected 3 h after the beginning of growth (see Fig. S6 in the supplemental material). The production kinetics suggests QS-controlled expression. Microscopy analysis of E. coli and *D. vulgaris* coculture in the presence of the *D. vulgaris* supernatant shows the loss of the interaction (Fig. 7). The sulfate respiration process is probably associated with the production of an antagonist or antagonists that inhibit the AI-2 activity. This production requires the presence of sulfate and is independent of the presence of C. acetobutylicum or E. coli. To date, a possible impact on global metabolism cannot be ruled out.

10.1128/mBio.02758-20.6 FIG S6 Kinetics of production of the inhibitors of AI-2 activity by *D. vulgaris* in Starkey medium. Supernatants from pure cultures of C. acetobutylicum and E. coli DH10B were taken after 30 h of culture in GY medium and filtered (0.2 μm). Then, the activity of AI-2 in the filtered samples was analyzed using the V. harveyi reporter strain BB170 in the absence (positive control, C) or in the presence of 5 μl of *D. vulgaris* supernatant (grown in Starkey medium) taken at different growth times (*n* = 3). Download FIG S6, TIF file, 1.1 MB. Copyright © 2021 Ranava et al. 2021 Ranava et al. This content is distributed under the terms of the Creative Commons Attribution 4.0 International license.

**FIG 8:**
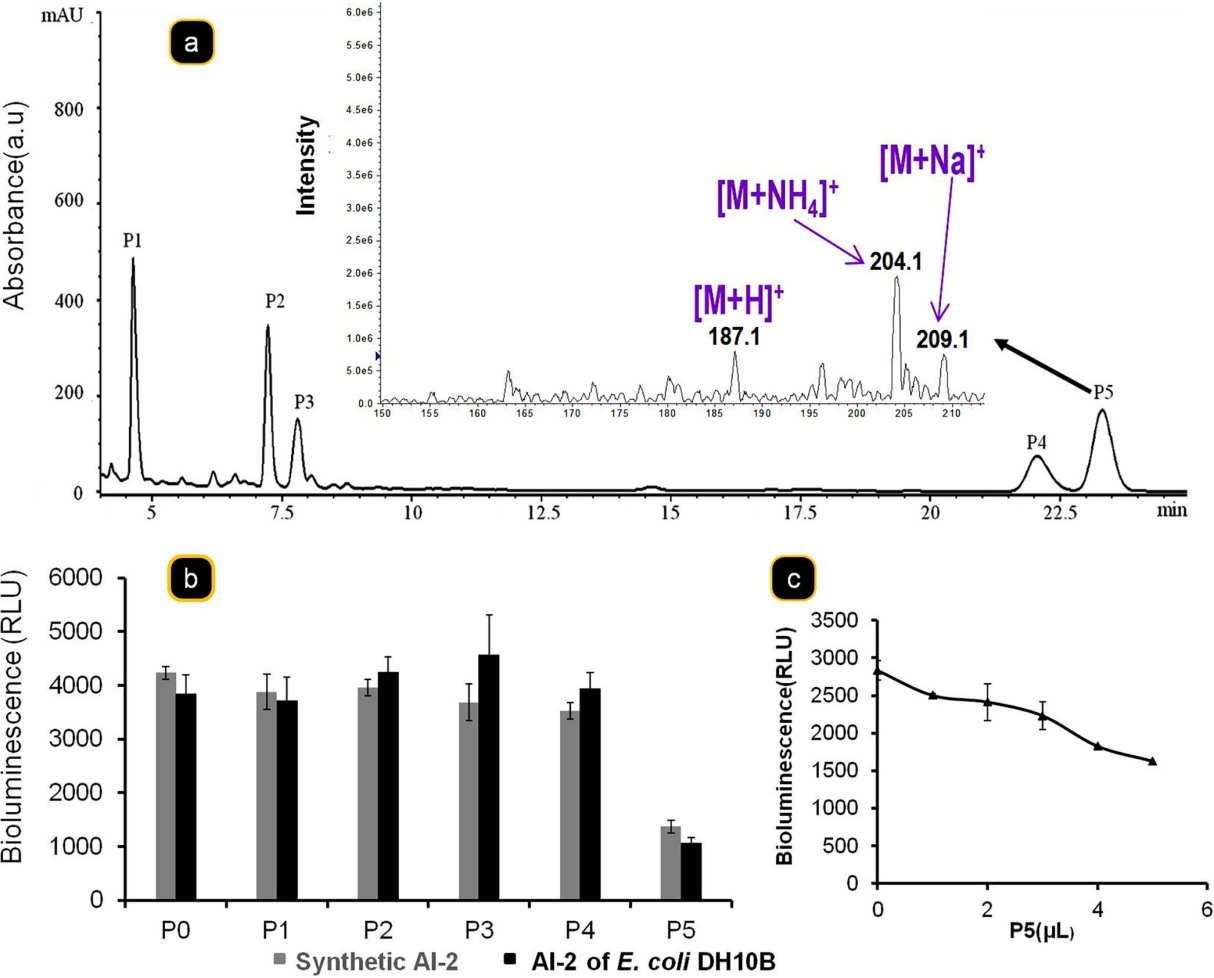
Identification of AI-2 inhibitor in *D. vulgaris* supernatant. (a) *D. vulgaris* strain was grown in Starkey medium during 30 h at 37°C, and the filtered supernatant was analyzed by RP-HPLC. The peak P5 (black arrow), which presents an AI-2 inhibitor activity, was analyzed mass spectrometry. (b) The AI-2 inhibitor activity of different peaks collected (P1 to P5) was determined on synthetic AI-2 [(*S*)-4,5-dihydroxy-2,3-pentandione; 2.5 μM] or on AI-2 produced by E. coli strain DHI0B grown on GY medium. (c) To test the hypothesis that the P5 can compete with AI-2, V. harveyi reporter strain BB170 was grown in AB medium (100 μl) supplemented with 500 nM synthetic AI-2 and in the presence of various quantities (0, 1, 2, 4, and 5 μl) of P5, and the bioluminescence was measured after 4 h.

To identify the compounds that interfere with the activity of AI-2, a cell-free supernatant of *D. vulgaris*, grown in Starkey medium for 30 h, was analyzed by high-pressure liquid chromatography (HPLC). All the major peaks (P1 to P5) were recorded, and their ability to inhibit AI-2 activity was determined on the supernatant of exponential-phase E. coli DH10B culture. Under our test conditions, the peak P5 has a stronger effect on AI-2 activity compared to peaks P1 to P4 (Fig. 8a and b). Peak P5 (Fig. 8a, indicated by black arrow) significantly inhibits the AI-2 activity in a dose-dependent manner (Fig. 8c). Interestingly, we observed similar inhibition of AI-2 activity in the presence of peak P5 when using an *in vitro*-synthesized AI-2. Since the addition of *D. vulgaris* supernatant does not impair the growth of V. harveyi (see Fig. S5), the bioluminescence reporter strain, this result suggests the direct impact of molecules produced by *D. vulgaris* on AI-2 activity. Peak P5 analysis by mass spectrometry reveals a compound that has a molecular mass equivalent to that of AI-2 (192.9 Da), suggesting a similar type of molecule that could act as a competitive inhibitor. Taken together, these data show that the V. harveyi AI-2 receptor LuxP can recognize the compound present in peak P5. However, it is still unclear whether this compound also binds to the AI-2 binding site or whether LuxP contains an independent binding site for this new compound. Different strategies for obtaining the structure did not succeed, probably because, as with AI-2, there is an equilibrium between various forms (37).

## DISCUSSION

The bacterial community established in batch culture by C. acetobutylicum plus *D. vulgaris* or by E. coli DH10B plus *D. vulgaris* and under conditions of nutritional stress for *D. vulgaris* (i) exchanges metabolites, allowing a satisfactory energetic state and growth of *D. vulgaris*, and (ii) is regulated by AI-2 molecules that allow physical and metabolic interactions in the coculture. Furthermore, in the presence of sulfate, *D. vulgaris* produces an AI-2 antagonist. Production of the antagonist is independent of the presence of C. acetobutylicum or E. coli and may prevent the formation of other consortia. Altogether, our studies show how QS molecules coordinate interactions between species and how this modulation follows environmental stress. Moreover, our findings illustrate how experiments with multiple species or synthetic ecological models can provide new insight into bacterial sociability.

The presence of C. acetobutylicum or E. coli DH10B allows *D. vulgaris* to be energetically viable despite the lack of sulfate. The observation that, without C. acetobutylicum or E. coli DH10B, *D. vulgaris* is not energized explains why the presence of one or the other of these two bacteria is indispensable for *D. vulgaris* growth. We previously demonstrated that proteins can be transferred via nanotube-like structures (18), which obviously could also be involved in the transfer of metabolites from one bacterium to another. However, we cannot exclude the possibility that these structures minimize the distance between the bacteria and create more or less a stable network that can prevent the diffusion of metabolites into the medium and thus increase their local concentration, allowing transfer by diffusion.

The presence of [^13^C]DNA from *D. vulgaris* in conditions of coculture with C. acetobutylicum using only [^13^C]glucose indicates that *D. vulgaris* can grow on metabolites produced by C. acetobutylicum and derived from [^13^C]glucose if the two bacteria are in close contact. Although *D. vulgaris* might just be using the metabolites produced by C. acetobutylicum and excreted to the culture medium, this is not supported by the observations that intercellular connections or at least in a very close environment appear indispensable to allow growth of *D. vulgaris* (18) since the presence of dialysis membrane that prevents the contact also prevents the growth. As in the coculture, in the absence of sulfate (electron acceptor) and under conditions of close contact between *D. vulgaris* and C. acetobutylicum, (i) *D. vulgaris* grows on the metabolites produced by C. acetobutylicum, (ii) *D. vulgaris* cannot grow by fermenting lactate because of inhibition by H_2_, and (iii) growth requires the existence of a respiratory metabolism. Thus, C. acetobutylicum may be acting as final electron acceptor through a mechanism that has not yet been elucidated.

How these kinds of interactions are initiated and controlled is at present poorly understood. Ben-Yehuda’s group showed that YmdB is involved in the late adaptive responses of B. subtilis in the early stage of nanotube development (12). In various types of cells, it is the cell undergoing stress that develops the formation of nanotubes, suggesting that this might be directly induced by stress and constitutes a defense mechanism (38), as apparently also occurs in this consortium.

Surprisingly, the role and the consequence of the QS molecules, well described in pure culture, are poorly understood in bacterial consortia, which are closer to what is found in nature. However, this topic has attracted the attention of researchers studying mixed cultures in bioreactors for treating wastewater. Although the real mechanism involved in QS regulation, in complex microbial consortia, remained to be elucidated, studies of this type have shed some light on these subjects (39, 40). Recently, one study investigated this question using a mathematical model to demonstrate how QS controls the population trajectories in synthetic consortium (41).

The QS molecule AI-2 is crucial for metabolic interaction between the two bacteria of the coculture, since there is no metabolic exchange in its absence. Thus, C. acetobutylicum produces a molecule with AI-2 activity that had not been described before; furthermore, the C. acetobutylicum gene *luxS_Ca_* can restore the AI-2 production by *E. coli* DH5α. Our results explain why E. coli can connect to other bacterial cells to exchange cytoplasmic molecules.

Some organisms can produce and sense AI-2, whereas others only sense the AI-2 signal (42, 43). *D. vulgaris* appears to be in the latter category, since it lacks the *luxS* gene but appears to sense AI-2. This suggests the presence of AI-2 receptors in *D. vulgaris.* However, no genes similar to *lsrB* (coding for LsrB, the protein receptor of AI-2 in enterobacteria) or to *luxP* (the gene coding for LuxP, the protein receptor of AI-2 in *Vibrionaceae*) are present in the genome of *D. vulgaris* (16). On the other hand, some bacteria can respond to an exogenous AI-2 signal (44, 45) despite lacking the genes *luxS*, *lsrB*, or *luxP.* Furthermore, two proteins, AibA and AibB, can bind AI-2 in Helicobacter pylori. Moreover, the deletion of the genes *hppA* and *modA* encoding these two proteins, induces deficiencies in chemotaxis and biofilm organization (46). By bioinformatic analysis we detected their homologues (*oppA* and *modA*, respectively) in *D. vulgaris*, and experiments are under way to study whether *D. vulgaris* internalizes AI-2, as preliminary results suggest. The issue of the cellular receptor of AI-2 thus remains an open question, and our results help to clarify this.

Although the original QS concept was focused on the detection of cell density for the regulation of gene expression, studies in microbial ecology suggest a wider function. For example, the efficient-sensing concept (47) assumes that the ecologically relevant function of AI-2 sensing is to preassess the efficiency of producing extracellular effectors or “public goods.” Cooperative genes regulated by QS molecules can also be sensitive to nutrient conditions, suggesting that metabolic information is integrated into the decision to cooperate. The correlation between QS and cell activity, rather than bacterial growth, has been recently underlined in *D. vulgaris* (32). AI-2 molecules are involved in the mechanism stimulating viable but noncultivable cell exits from dormancy, perhaps signaling to dormant cells when conditions are now favorable for growth (48, 49). This supports the idea that AI-2-dependent signaling reflects the metabolic state of the cell and can function as a proxy for the production of effectors, such as enzymes, or the formation of nanotubes. Integrating metabolic information with QS offers a possible mechanism to prevent cheating, since cells can only cooperate when they have the appropriate nutritional resources to do so, reducing the cost of cooperation to the individual cell (50).

We demonstrate the quenching of the AI-2 activity by a quorum-quenching (QQ) molecule produced by *D. vulgaris* in the presence of lactate and sulfate. QQ has been suggested to be achieved in three ways: (i) by blocking the synthesis of autoinducers, (ii) by interfering with signal receptors, and (iii) by degrading the autoinducers (51–54). Since we show competition between AI-2 molecules present in the C. acetobutylicum or E. coli supernatant, or even between synthetic AI-2 and an AI-2 quencher, a small molecule present in the *D. vulgaris* supernatant, we can exclude the first and the third hypotheses. Only a few AI-2 interfering mechanisms have been reported, and most of them include synthetic molecules as a quencher (37, 55, 56). It has been proposed that C1 alkyl, an analogue of AI-2, could compete with AI-2 for the LsrR transcriptional regulator in the Lsr system (37, 57), and the presence of the competitor is linked to a decrease in AI-2 production. Since AI-2 consists of a group of molecules in equilibrium and is not a unique defined structure (37), analogy with enzymes and alternative substrates suggests that different types of AI-2 molecules may interact with a receptor, with only some of them inducing a response. We cannot discard the possibility that the QQ molecule identified in *D. vulgaris* supernatant could bind to an AI-2 receptor in C. acetobutylicum and induce an effect at the level of gene transcription that could be translated into metabolic modification. Moreover, we also cannot discard the possibility that the QQ molecule identified represents a QS signal for *D. vulgaris*.

Our analysis provides new insights into metabolic prudence (58) and bacterial communication and how metabolic signals influence social behavior, but many details of its molecular implementation remain to be discovered. Which proteins detect the metabolic signals? How do they interact with QS regulation at the molecular level? One should also be cautious in using the word “signaling” because every change in a living organism affects every other and thus acts as a signal of some kind (59, 60). In all of these studies, it is important to keep in mind the ecological context, but the analysis of how the components of an ecological system influence one another has barely begun (61). We can now see in these microbial communities established, thanks to QS molecules, the preliminary steps in the evolutionary pathway of multicellular organisms and eukaryotes.

## MATERIALS AND METHODS

### Media and growth conditions.

Strains were grown to steady state in Hungate tubes under anaerobic conditions, in Luria-Bertani (LB) medium for E. coli DH10B and DH5α, in Starkey medium (containing lactate and sulfate) for D. vulgaris (62), and in 2YTG medium for C. acetobutylicum (63) to an optimal absorbance of 0.6. The growth medium (glucose-yeast extract [GY] medium) used to study the consortium was prepared with glucose (14 mM) and 0.1% yeast extract and supplemented with the similar inorganic nutrients used for the Starkey preparation (but with MgCl_2_ instead of MgSO_4_). GY medium was inoculated with either washed *D. vulgaris*, C. acetobutylicum, or E. coli or with a combination of different strains to constitute an artificial consortium in a 1:1 ratio according to the absorbance at 600 nm. *D. vulgaris* is not able to grow alone in GY medium (18). In some cases, the growth medium was supplemented with 5 or 10 mM lactate and/or 5 or 10 mM sulfate. The experiments were carried out at least in triplicate.

### Construction of *E. coli* DH5α (*luxS_Ca_*) strain.

The *luxS* open reading frame (corresponding to gene *CA_C2942*) was amplified using the genome of C. acetobutylicum as a template and the oligonucleotide primers LuxS (5′-GAAACCGGTAAAACAAAGGAGGACGTTTATGGAAAAAATCGCAAGTTTTACTG-3′) and LuxS-RevpB (5′-GATCGATGGTACCTTATCAGTGGTGGTGGTGGTGGTGCTCTGGATAATTTAATCTATCTTCAGATATG-3′). For PCR amplification, *luxS* was digested and introduced into the AgeI and KpnI sites of the pBGF4 plasmid under the control of a hydrogenase constitutive strong promoter (64) to obtain pRD4 plasmid. The DNA sequence was analyzed by DNA sequencing (Cogenics). Next, pRD4 was transformed in E. coli DH5α to obtain the E. coli DH5α(*luxS_Ca_*) strain. As a negative control, E. coli DH5α was also transformed with empty plasmid pBGF4.

### Labeling of *D. vulgaris* with calcein-acetoxymethyl ester.

The labeling of *D. vulgaris* cells by calcein was carried out as described by Benomar et al. (18). Briefly, *D. vulgaris* cells were grown in Starkey medium (62) under anaerobic conditions, and then exponentially growing *D. vulgaris* cells (5 ml) were harvested at room temperature by centrifugation at 4,000 × *g* for 10 min, washed twice with Starkey medium, and resuspended in 5 ml of fresh Starkey medium. Next, 100 μl of calcein-AM ester (1 mg/ml in dimethyl sulfoxide [DMSO]; Sigma) was then added to the medium. The suspension was incubated in the dark at 37°C for 2 h under anaerobic conditions. The cells were subsequently harvested and washed three times in fresh, dye-free GY medium and used in the exchange experiments.

### Labeling of *E. coli* DH5α and *E. coli* DH10 with mCherry.

Labeling was carried out by transforming E. coli DH5α and E. coli DH10 with pRSET-B mCherry (Addgene, plasmid 108857).

### Exchange of cytoplasmic molecules between *D. vulgaris* and *E. coli*.

To study the exchange of molecules between the two bacteria, *D. vulgaris* labeled with calcein was mixed with several strains of E. coli labeled with mCherry: E. coli DH10B strain, a producer of the AI-2 molecule; E. coli DH5α, a nonproducer of the AI-2 molecule; and DH5α(*luxS_Ca_*). In some cases, unlabeled cells of *D. vulgaris* were mixed with E. coli labeled with mCherry. The mixture was diluted in 5 ml of fresh GY medium and put in a tube containing a coverslip and incubated at 37°C for 20 h. The coverslip was removed after 20 h of growth, and bacterial cells attached to coverslip were visualized by fluorescence confocal microscopy, as previously described by Benomar et al. (18).

### AI-2 activity assay.

The AI-2 activities of cell-free culture supernatants were measured by using V. harveyi reporter strain BB170, as described by Bassler et al. (65). Briefly, an overnight culture of V. harveyi (grown for 16 h in AB medium) was diluted 1/5,000 in fresh AB medium (300 mM NaCl, 50 mM MgSO_4_, 2% [wt/vol] Casamino Acids, 10 mM potassium phosphate [pH 7], 1 mM l-arginine, 1% [wt/vol] glycerol). The diluted cells (90 μl) were added to 96-well plates (Corning) containing 10 μl of a cell-free culture supernatant of E. coli, D. vulgaris, or C. acetobutylicum obtained after centrifugation and filtration through 0.2-μm-pore-size membranes to remove bacterial cells or synthetic AI-2 molecule. The microtiter plate was incubated at 30°C with shaking at 160 rpm, and the bioluminescence was measured each hour over the course of 4 to 6 h using a Tecan GENioS plate reader (Tecan). The AI-2 activity is reported as the induction of bioluminescence, which is expressed in relative light units (RLU). The reported values represent the average bioluminescence stimulated by three independent preparations of cell-free culture fluids or synthetic AI-2 molecule. Similar experiments were performed in the presence of various amounts of *D. vulgaris* culture supernatant in Starkey medium. Cell-free culture supernatants of E. coli, *D. vulgaris*, or C. acetobutylicum were obtained at the exponential phase (optical density at 600 nm [OD_600_] ≈ 0.7/0.8).

### Use of RedoxSensor Green as a probe for active respiration in *D. vulgaris*.

RedoxSensor Green (RSG; Backlight RedoxSensor Green Vitality kit; Life Technologies) was used to assess cellular respiration activity of *D. vulgaris. D. vulgaris* cells were taken from Starkey medium culture in mid-log phase, washed twice, and starved by incubation in GY medium (without lactate and sulfate) for 20 h at 37°C. After starvation, *D. vulgaris* cells were harvested at room temperature by centrifugation at 4,000 × *g* for 10 min, washed twice with GY medium, and diluted in 5 ml of fresh GY medium containing 1 μM RSG reagent. The cultures were supplemented with 10 mM lactate and 10 mM sulfate or not, and bacterial cells were imaged by fluorescence microscopy after incubation at 37°C for 20 h. After 20 h of incubation at 37°C, the coculture was left for 5 min in contact with FM4-64 to visualize the bacterial membrane and the C. acetobutylicum, which was not labeled at the beginning of the experiment. Also, the effect of the electron transport chain uncoupler CCCP on RSG fluorescence was verified to further confirm the redox sensing functionality of RSG in *D. vulgaris*, as previously reported for other bacteria (9).

### Analysis and purification of the antagonist AI-2 compounds from *D. vulgaris*.

*D. vulgaris* cells were grown in Starkey medium under anaerobic conditions for 30 h at 37°C. The culture was then centrifuged at 10,000 rpm for 5 min and filtered through 0.2-μm membranes to remove the cells. The cell-free supernatants were stored at –20°C or immediately analyzed by HPLC. The analysis was carried out on an Agilent 1200 HPLC system equipped with a UV detector and a refractometer (Agilent Technologies). Separation (20- to 50-μl portions of samples were injected) was achieved on an Agilent Poroshell EC-C18 reversed-phase column (C_18_, 4.6 by 150 mm, 2.7 μm) set at 30°C. The compounds were eluted with 8% solution A (acetonitrile plus 1% [vol/vol] formic acid) and 92% of solution B (deionized water plus 1% [vol/vol] formic acid) at a flow rate of 0.6 ml/min. Purified compounds were directly used for bioluminescence assay or lyophilized and stored at –20°C.

### Mass spectrometry analysis.

Analyzes of samples were performed with a 3200 QTRAP mass spectrometer (Applied Biosystems Sciex) equipped with a pneumatically assisted atmospheric pressure ionization source. The sample was ionized in positive electrospray mode under the following conditions: electrospray voltage (ISV), 5,500 V; orifice voltage (OR), 10 V; and nebulizing gas pressure (air), 10 lb/in^2^. The sample was also ionized in negative electrospray mode under the following conditions: ISV, 4,500 V; OR, 10 V; and nebulizing gas pressure (air), 10 lb/in^2^. Mass spectra were obtained with a quadrupole analyzer.

Samples are dissolved in 300 μl of acetonitrile and then diluted 1/10 in a 3 mM methanol solution of ammonium acetate. Sample solutions are introduced into the ionization source by infusion (Harvard apparatus syringe pump) at a flow rate of 10 μl/min.

### Carbon exchange by stable isotope probing.

The stable isotope probing method described by Neufeld et al. (66) and derived from Meselson and Stahl (69) was slightly modified. GY medium was prepared with d-glucose-^13^C6 (14 mM) from Cortecnet and 0.1% yeast extract (^12^C), supplemented with similar inorganic nutrients, as used for the Starkey preparation (MgCl_2_ instead of MgSO_4_) and N_2_ in the headspace. ^13^C-GY medium was inoculated (10%) with either washed C. acetobutylicum enriched in ^13^C via 26 subcultures or *D. vulgaris* ^12^C at exponential growth phase, or with the two strains, to constitute an artificial consortium in a 1:1 ratio according to the absorbance at 600 nm. At the end of the exponential phase, genomic DNA was extracted from the cell pellets using a NucleoBond AXG20 kit (Macherey-Nagel), and the DNA purity and concentration were determined with a NanoDrop 2000c spectrophotometer (Thermo Scientific).

To separate labeled/heavier ([^13^C]DNA) from unlabeled/lighter ([^12^C]DNA) content, density gradient centrifugation was performed in 5.1-ml quick-seal tubes in an NVT 65.2 rotor (near vertical) using an Optima L-90K centrifuge (Beckman Coulter). CsCl medium with an average density of 1.72 g/ml was loaded with 6 μg of total extracted DNA. After centrifugation at 20°C for 66 h at 41,500 rpm (169,000 × *g*), each gradient (^13^C-labeled C. acetobutylicum plus ^12^C-unlabeled*-D. vulgaris* and ^12^C-unlabeled C. acetobutylicum plus ^12^C-unlabeled *D. vulgaris*) was fractionated from the bottom to the top by displacement with mineral oil. The DNA in each fraction was precipitated as described by Neufeld et al. (66).

The presence and relative amount of DNA in fraction 28 (see Fig. S3 in the supplemental material) from each bacterium were monitored by qPCR. The primers used for the qPCR (*dsrA* and *endoG*) are listed in Table S1 in the supplemental material. The reaction was performed with a Bio-Rad SsoFast Eva Green Super Mix 2× kit and was carried out in a Bio-Rad CFX 96 as follows: 2 min and 30 s at 98°C for initial activation of enzymes, followed by 45 cycles of 5 s at 98°C, 10 s at 58°C, and 2 s at 72°C. These experiments were performed in triplicate.

10.1128/mBio.02758-20.7 TABLE S1 Oligonucleotide primers used for quantitative PCR. *drsA* and *endoG* are specific genes for *D. vulgaris* and C. acetobutylicum, respectively. Download Table S1, DOCX file, 0.02 MB. Copyright © 2021 Ranava et al. 2021 Ranava et al. This content is distributed under the terms of the Creative Commons Attribution 4.0 International license.

### AI-2 synthesis.

**FIG 9:**
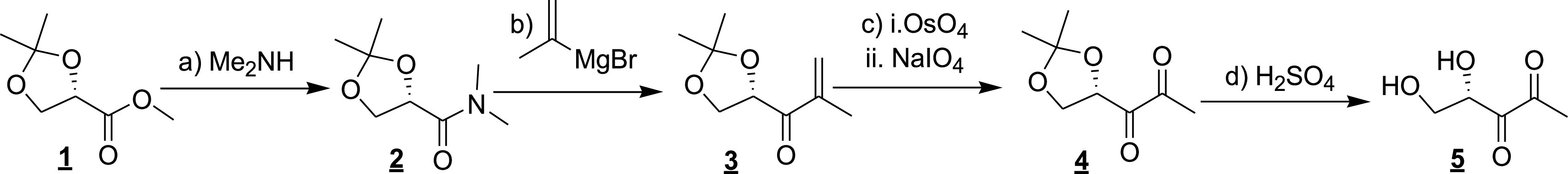
Four-step sequence used to obtain AI-2. (a) Me_2_NH, EtOH, 0°C to room temperature (RT), 48 h, 86%; (b) *i*-propenyl-MgBr, Et_2_O 48 h, 46%; (c, i) OsO_4_, NMO, CH_3_COCH_3_: H_2_O; (c, ii) NaIO_4_, MeOH: H_2_O, RT, 30 min, 25% over two steps; (d) H_2_SO_4_, D_2_O: *d*_6_-DMSO, 0°C, 1 h.

AI-2 was obtained in a four-step sequence (Fig. 9), starting from the commercially available methyl (*S*)-(–)-2,2-dimethyl-1,3-dioxolane-4-carboxylate and adapting the reported procedures (67, 68).

First, the methyl ester (see Fig. 9, compound 1) was transformed into the amide (compound 2) by reacting with dimethylamine in ethanol (EtOH). The reaction of amide (compound 1) with isopropenyl magnesium bromide gives olefin (compound 3). Dihydroxylation of olefin (compound 3) with catalytic osmium tetroxide in the presence of *N*-methylmorpholine-*N*-oxide and subsequent cleavage of generated diol with NaIO_4_ produced ketone (compound 4). Finally, the hydrolysis of dioxolane ring in acidic condition yields AI-2 (compound 5) and its cyclic anomeric products. The spectral data were consistent with those previously reported. The details of each step are detailed below.

**FIG 10:**
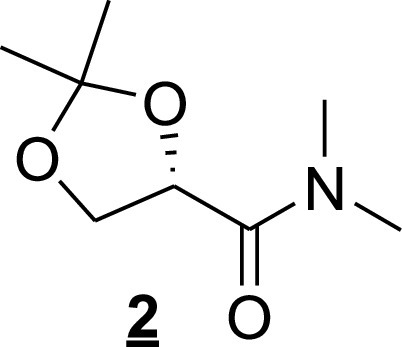
Amide (compound 2) structure.

For *N*,*N*-dimethyl (*S*)-α,β-isopropylidene glyceramide (compound 2 [Fig. 10]), methyl (*S*)-α,β-isopropylidene glycerate (3 g, 18.5 mmol) was added to the solution containing dimethylamine (20 ml, 30 vol% in ethanol) at 0°C. The mixture was stirred for 24 h. After adding an additional 10 ml of dimethylamine solution, the stirring was continued for a further 24 h. The volatile compounds were evaporated, and the residue was purified by column chromatography to yield 2.8 g of amide (compound 2): 86%, colorless liquid; *R_f_* = 0.2 (pentane/ethyl acetate, 3:2); ^1^H NMR (400 MHz, CDCl_3_) ∂ = 4.62 (t, *J *=* *6.65 Hz, 1H), 4.31 (dd, *J *=* *8.28, 6.53 Hz, 1H), 4.07 (dd, *J *=* *8.53, 6.78 Hz, 1H), 3.05 (s, 3H), 2.90 (s, 3H), 1.34 (s, 6×H).

**FIG 11:**
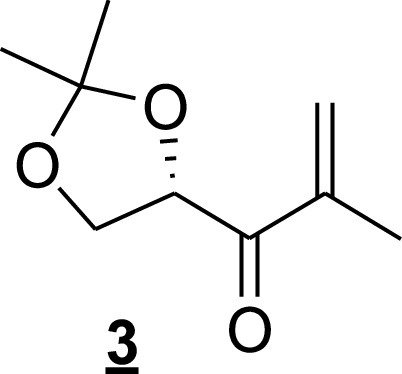
Olefin (compound 3) structure.

For (*S*)-4-methacryloyl-2,2-dimethyl-1,3-dioxolane (step 3), 21.0 ml of a 0.5 M solution of isopropenyl magnesium bromide in THF was added to a solution of amide (compound 2 [Fig. 10]; 1.74 g, 10 mmol) in anhydrous diethyl ether (10 ml) at 0°C under an argon atmosphere, followed by stirring for 15 min. The reaction mixture was quenched with 1 M HCl and extracted three times with diethyl ether, and the combined organic phase was dried over Na_2_SO_4_ and evaporated to dryness. The residue was purified by chromatography on SiO_2_ to afford compound 3 (0.8 g, 46% [Fig. 11]): *R_f_* = 0.33 (pentane/ethyl acetate, 9:1); ^1^H NMR (300 MHz, CDCl_3_) δ = 6.06 (s, 1H), 5.91 (d, *J *=* *1.38, 1H), 5.08 (dd, *J *=* *7.26,6.03, 1H), 4.22 (dd, *J *=* *8.34, 7.4, 1H), 4.09 (dd, *J *=* *8.43,5.94, 1H), 1.89 (s, 3H), 1.41 (s, 6×H).

**FIG 12:**
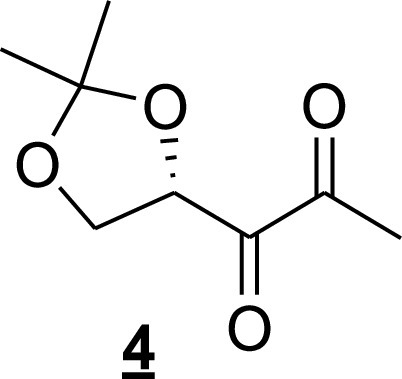
Ketone (compound 4) structure.

For 1-(2,2-dimethyl-[1,3]dioxolan-4-yl)-propane-1,2-dione (compound 4 [Fig. 12]), the alkene (compound 3 [Fig. 11]; 40 mg, 0.23 mmol) was dissolved in a mixture of acetone and water (4 ml/1 ml). *N*-methylmorpholine-*N*-oxide monohydrate (39 mg, 0.23 mmol) was added slowly, and the reaction mixture was stirred at room temperature for 10 min. Then, a 4% aqueous solution of osmium tetraoxide (0.1 ml, 0.011 mmol) was added to the above reaction mixture, followed by stirring overnight at room temperature. The mixture was quenched with Na_2_SO_3_ and extracted with dichloromethane. The organic layer was dried (Na_2_SO_4_) and filtered, and the filtrate was concentrated under reduced pressure. The crude diol was used in the next step as such. To the diol in methanol (3.5 ml) and water (1.5 ml) was added sodium periodate (0.146 g, 0.69 mmol), followed by stirring at room temperature for 30 min. The reaction mixture was diluted with water and extracted with dichloromethane. The organic layer was dried (Na_2_SO_4_) and filtered, and the filtrate was concentrated under reduced pressure. The residue was purified by chromatography on SiO_2_ to yield compound 4 (Fig. 12; 15.9 mg, 40% over two steps). *R_f_* = 0.65 (pentane/ethyl acetate, 6:4); ^1^H NMR (400 MHz, CDCl_3_) δ *=* 5.14 (dd, *J *=* *7.8, 5.8, 1H), 4.37 (t, *J *=* *8.4, 1H), 4.0 (dd, *J *=* *9.00, 5.4, 1H), 2.39 (s, 3H), 1.47 (s, 3H), 1.42 (s, 3H).

**FIG 13:**
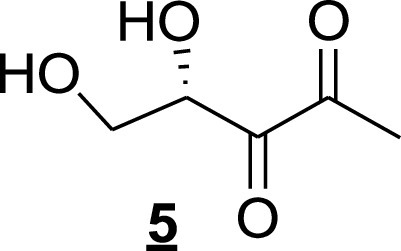
AI-2 (compound 5) structure.

To produce (*S*)-4,5-dihydroxy-pentane-2,3-dione (step 5), H_2_SO_4_ (0.005 ml) was added to a solution of diketone compound 4 (3.0 mg, 0.017 mmol) in D_2_O (0.5 ml) and *d*_6_-DMSO (0.2 ml) under an argon atmosphere, followed by stirring at room temperature for 1 h. The reaction mixture was quenched with 5 mg of Na_2_CO_3_ and filtered to obtain a 0.0249 M solution of AI-2 (compound 5 [Fig. 13]) and its cyclic anomeric products in D_2_O and *d*_6_-DMSO. The spectral data were consistent with those previously reported: ^1^H NMR (300 MHz, D_2_O): 4.25 (t, *J* = 6.4 Hz, 1H), 4.10 to 4.02 (m, 2H), 3.93 (dd, *J* = 3.4, 5.6 Hz,1H), 3.76 to 3.67 (m, 2H), 3.84 to 3.81 (m, 1H), 3.53 (dd, *J* = 7.5, 11.6 Hz, 1H), 3.45 (dd, *J *=* *5.4, 9.2 Hz, 1H), 2.27 (s, 3H), 1.34 (s, 3H), 1.31 (s, 3H). HRMS (ESI-TOF) *m/z*: [M-H], calculated for C_5_H_7_O_4_, 131.0350; found, 131.0354.

### Imaging and quantification of microscopy images.

Cells attached to the coverslip were observed by the Confocal Olympus FV1000 microscope (Japan) using the UPLSAPO 100× objective (oil immersion). The excitation and emission wavelengths are, respectively, 488 and 515 nm for calcein and RSG and 558 and 583 nm for mCherry. The laser beams were activated in sequential mode to avoid fluorescence overlaps. The microscopy fields analyzed were obtained from three biological replicates obtained independently containing between 100 and 150 cells. These fields were chosen according to the local cell density. Since we made a morphological discrimination to identify the different bacterial species, we scanned the entire coverslip to find microscopy fields with small clumps of bacteria where the morphology of each was observable. They were counted manually using ImageJ software.

### Statistical analysis.

All the data were obtained from at least three biologically independent replicates. Data analysis was performed using the R statistical analysis software v3.6.0. Statistical data were accomplished using R base function “aov” for the Tukey test. The Tukey test was used because it is a statistical test to perform a multiple comparison in one step. Data were compared to the base condition, and significances were estimated based on *P* values (*, *P* < 0.05; **, *P* < 0.01; ***, *P* < 0.001). In all cases, *P* values of <0.05 were considered significant. For details regarding statistical tests, see Data Set S1 in the supplemental material.

10.1128/mBio.02758-20.8 DATA SET S1 Statistical data analysis. The statistical analyses were performed using the R software v3.6.0. This table presents the means and the SD calculated for each set of data (sheet 1). *P* values were obtained by performing a Tukey test compared to control conditions (sheet 2). SD, standard deviation. Download Data Set S1, XLSX file, 0.02 MB. Copyright © 2021 Ranava et al. 2021 Ranava et al. This content is distributed under the terms of the Creative Commons Attribution 4.0 International license.
