# Supplementary figures and images for: Metabolic Exchange and Energetic Coupling between Nutritionally Stressed Bacterial Species: Role of Quorum-Sensing Molecules
Source: mBio. 2021 Jan 19;12(1):e02758-20. doi: 10.1128/mBio.02758-20 (PMC7845633; doi:10.1128/mBio.02758-20)

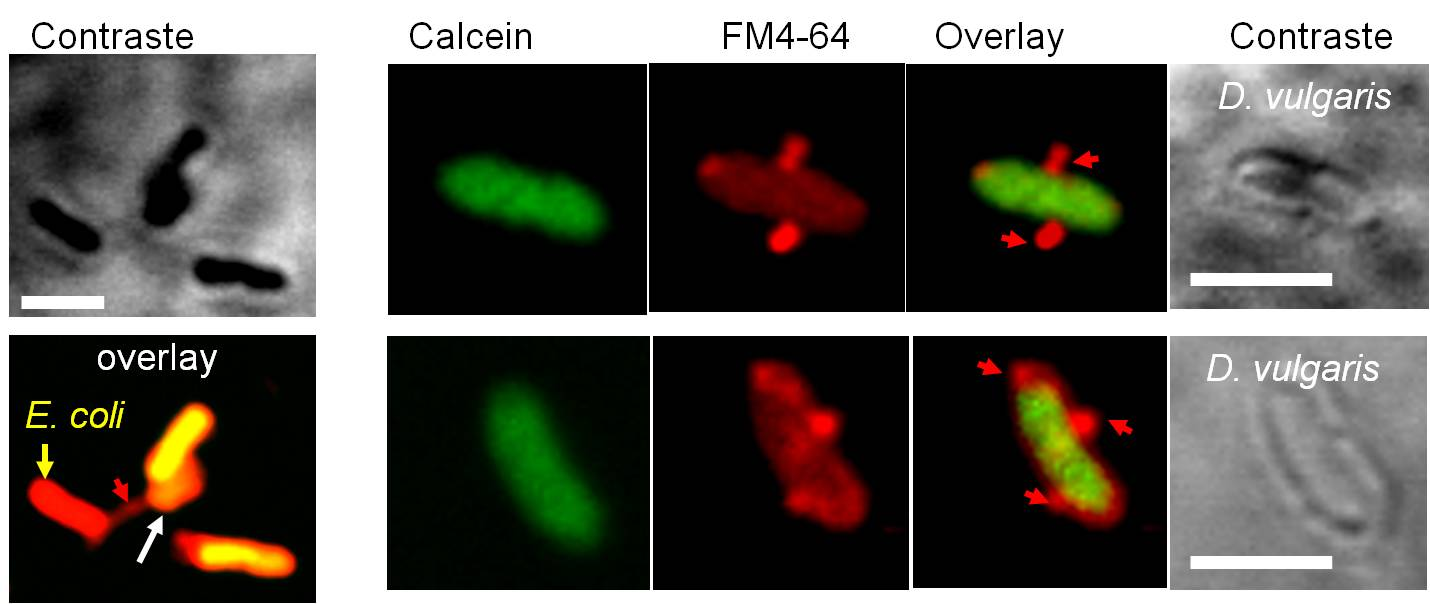

Supplement: FIG S1 [file mBio.02758-20-sf001.tif]

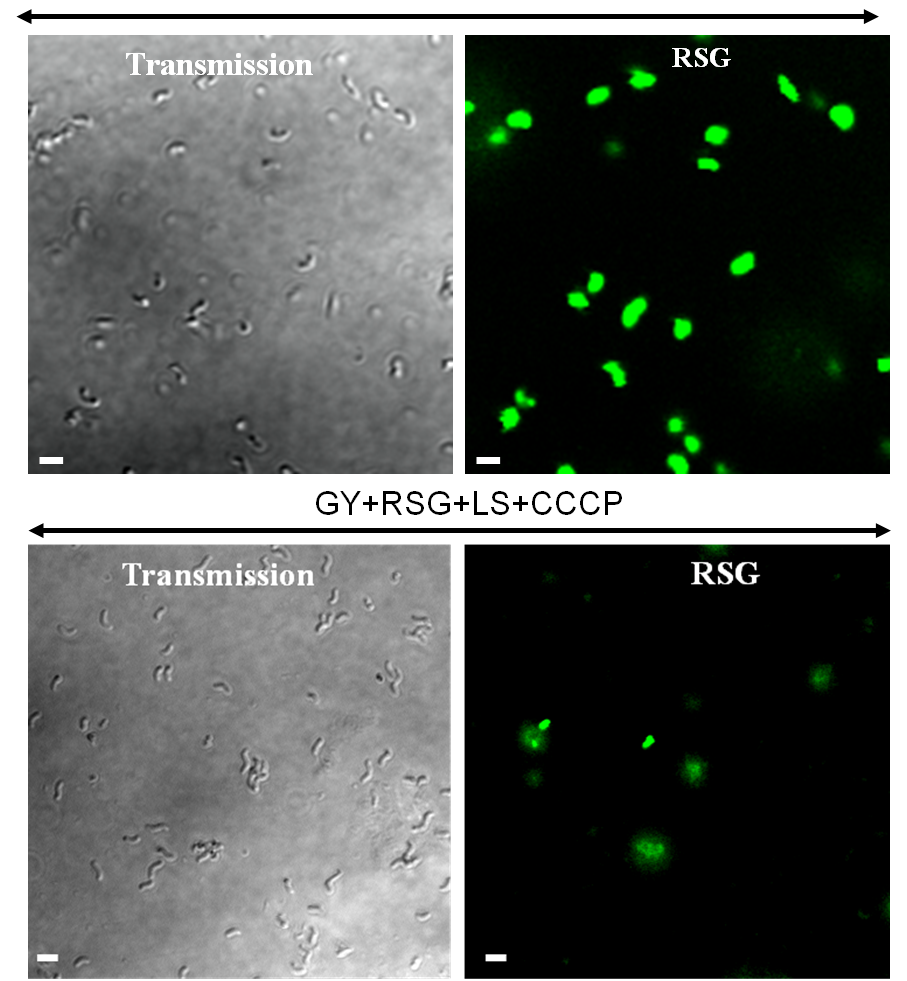

Supplement: FIG S2 [file mBio.02758-20-sf002.tif]

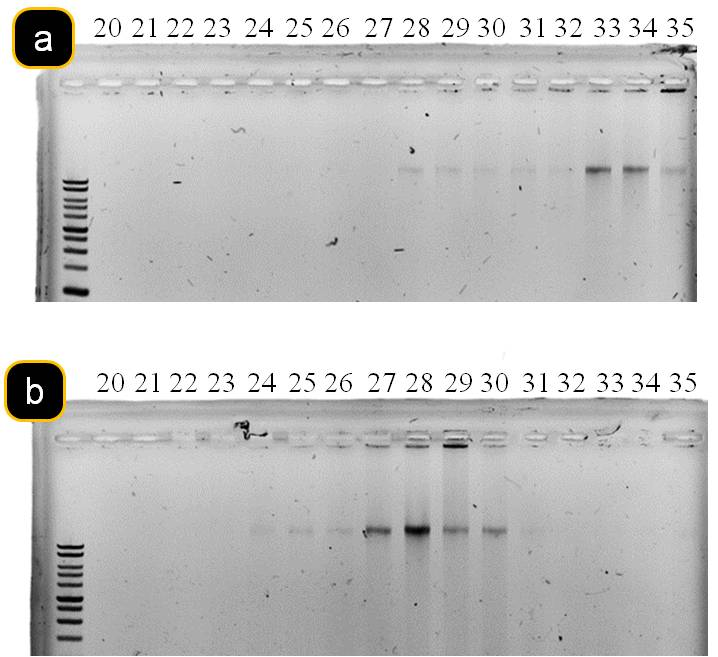

Supplement: FIG S3 [file mBio.02758-20-sf003.tif]

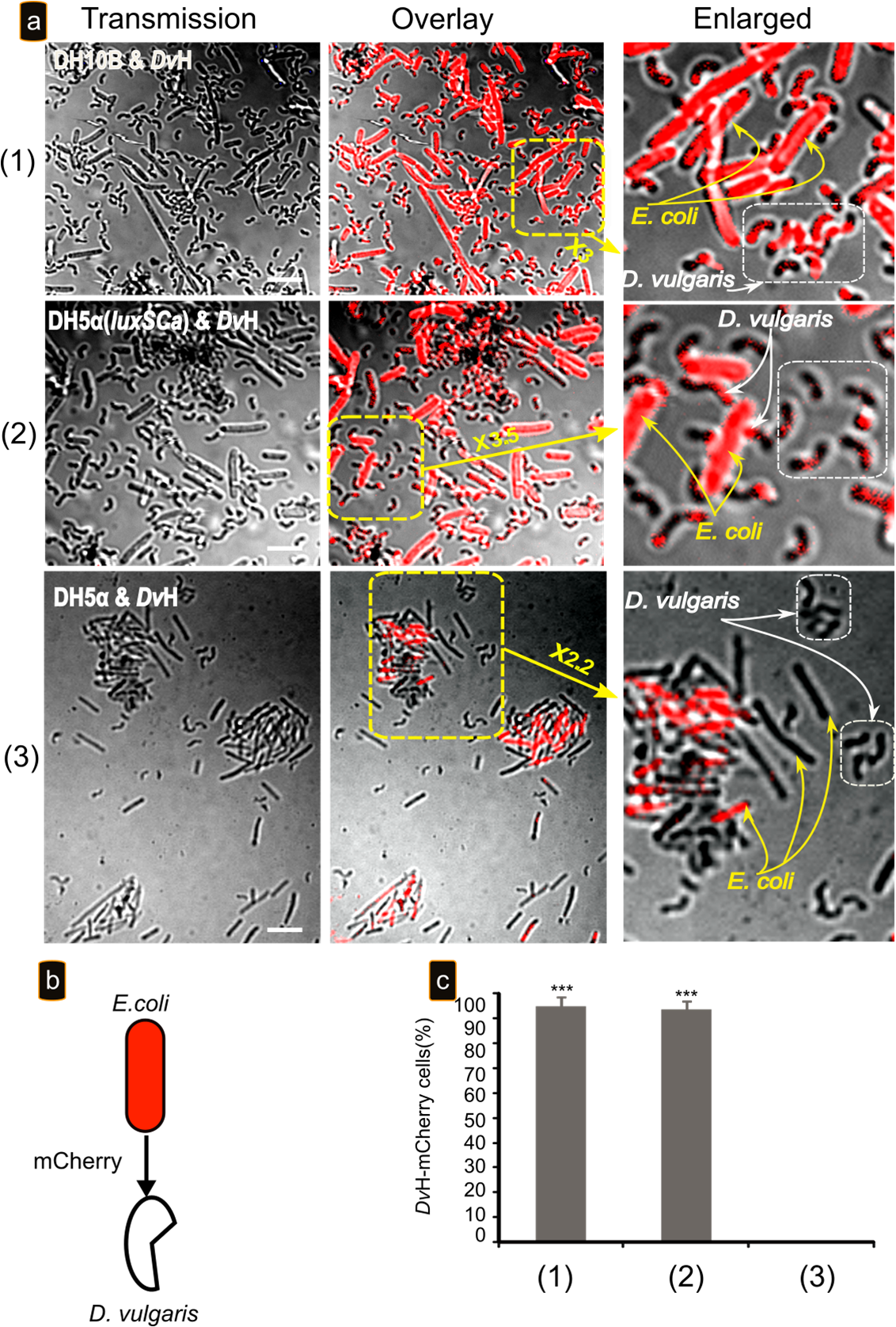

Supplement: FIG S4 [file mBio.02758-20-sf004.tif]

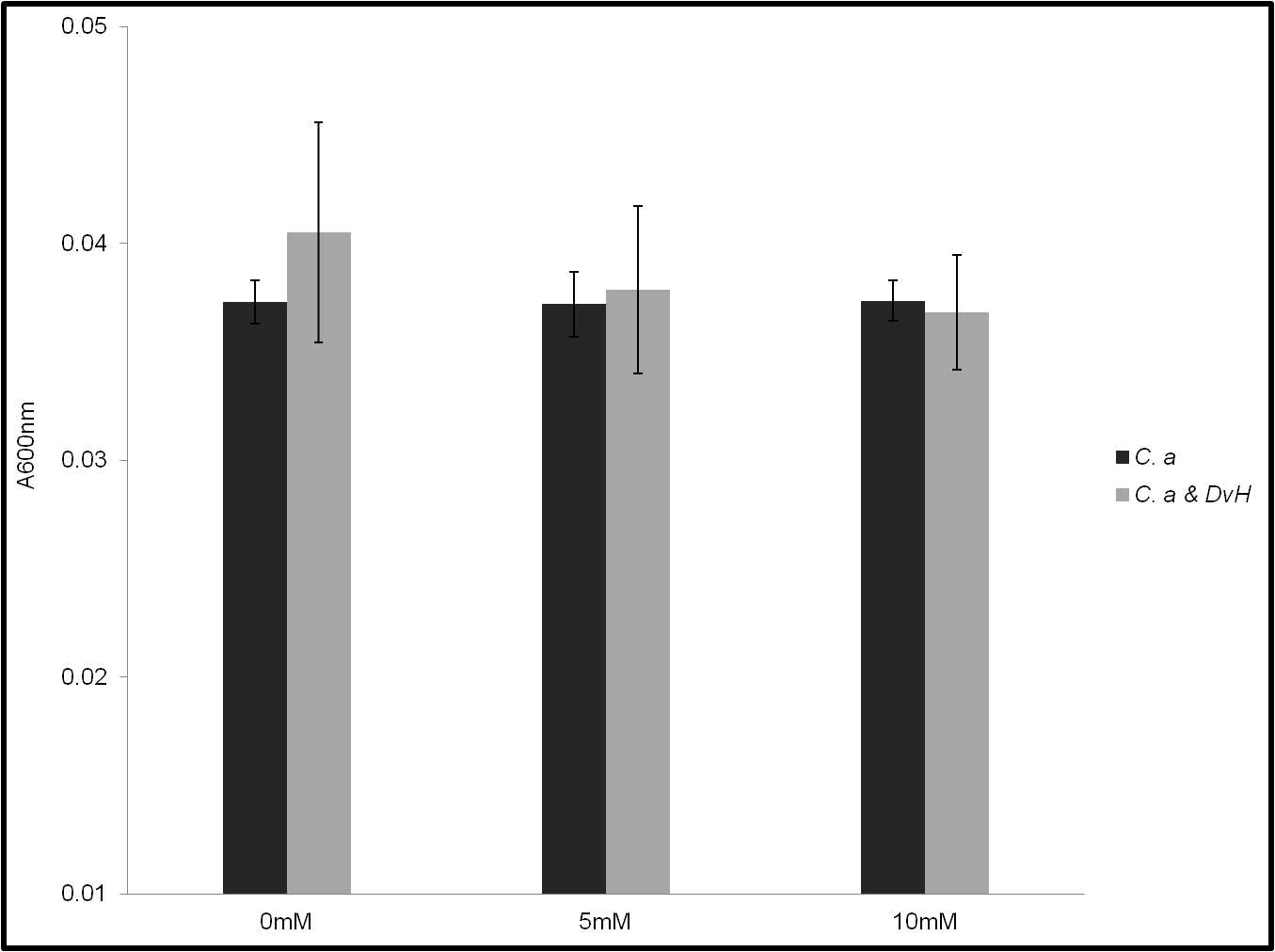

Supplement: FIG S5 [file mBio.02758-20-sf005.tif]

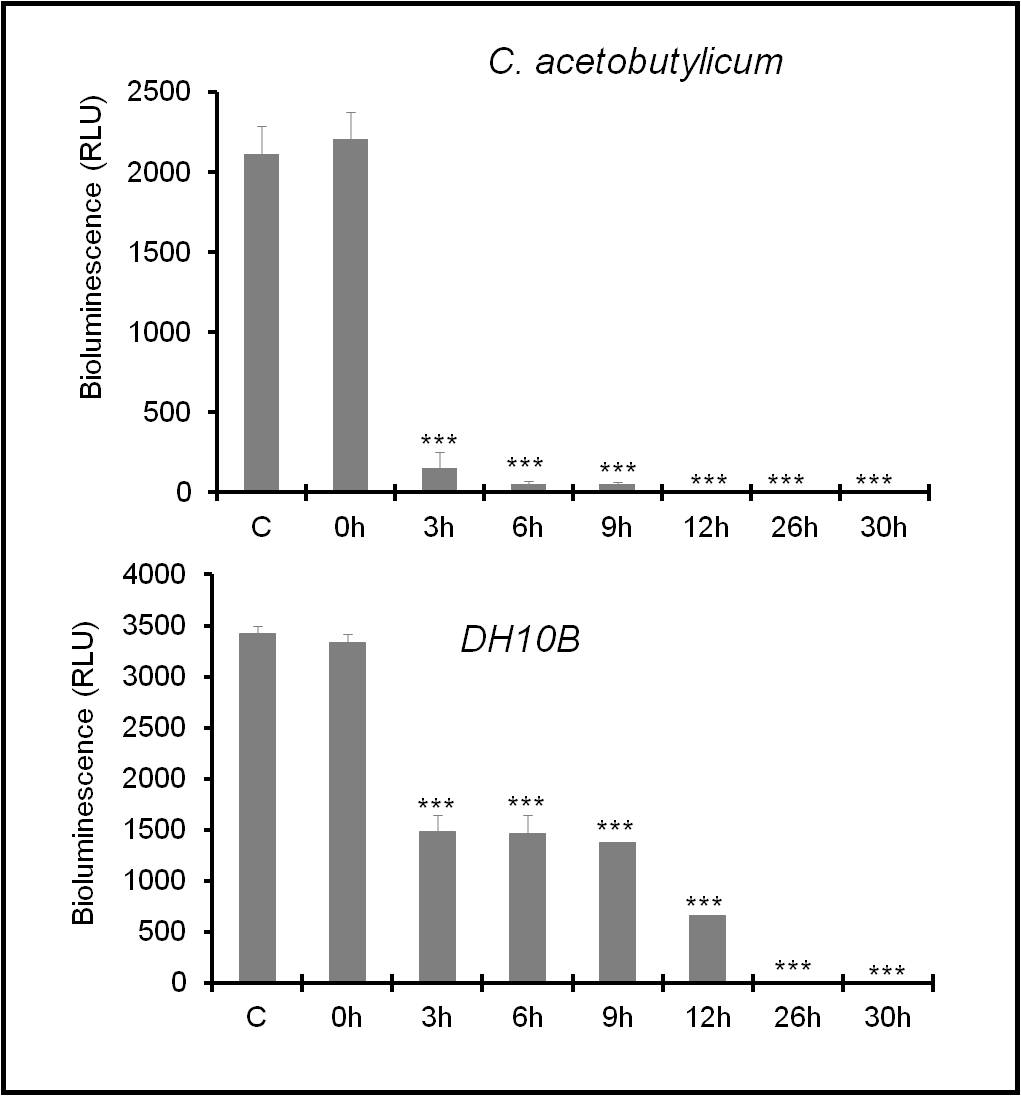

Supplement: FIG S6 [file mBio.02758-20-sf006.tif]
