# Supplementary material for: Metabolic Exchange and Energetic Coupling between Nutritionally Stressed Bacterial Species: Role of Quorum-Sensing Molecules
Source: mBio. 2021 Jan 19;12(1):e02758-20. doi: 10.1128/mBio.02758-20 (PMC7845633; doi:10.1128/mBio.02758-20)
Supplement: TABLE S1 [file mBio.02758-20-st001.docx]

**Supplementary Table 1: Oligonucleotide primers used for quantitative PCR.**

| **Strains** | \|  \| **Genes** \|  \| \| --- \| --- \| --- \| | **Sequences 5’- 3’** |
| --- | --- | --- | --- | --- | --- |
| \| *D. vulgaris* \| \| --- \| | \| *dsrA-fwd*  *dsrA-rev* \| \| --- \| \|  \| | \| GAATTCGCCTGCTACGACTC  TCCTTCCAGGTACCGATGAC \| \| --- \| |
| \| *C. acetobutylicum* \| \| --- \| | \| *endoG-fwd*  *endoG-rev* \| \| --- \| | \| ATGCGGCTACAGCTGAACT  ATTCTTTGCACCGGTGTCTC \| \| --- \| |
